# Potential of Curcumin and Its Analogs in Glioblastoma Therapy

**DOI:** 10.3390/antiox14030351

**Published:** 2025-03-18

**Authors:** Agnieszka Nowacka, Ewa Ziółkowska, Wojciech Smuczyński, Dominika Bożiłow, Maciej Śniegocki

**Affiliations:** 1 Department of Neurosurgery, Collegium Medicum in Bydgoszcz, Nicolas Copernicus University in Toruń, ul. Curie Skłodowskiej 9, 85-094 Bydgoszcz, Poland; 2 Department of Pediatrics, Washington University School of Medicine, St. Louis, MO 63110, USA; e.ziolkowska@wustl.edu; 3 Department of Physiotherapy, Collegium Medicum in Bydgoszcz, Nicolas Copernicus University in Toruń, ul. Techników 3, 85-801 Bydgoszcz, Poland; 4 Anaesthesiology and Intensive Care Clinical Ward, The 10th Military Research Hospital and Polyclinic, ul. Powstańców Warszawy 5, 85-681 Bydgoszcz, Poland

**Keywords:** curcumin, glioblastoma, GBM, anti-cancer, glioblastoma therapy, oxidative stress, Pl3K/Akt, NF-κB, JAK/STAT, p53, MAPK, Shh, radiosensitization, curcumin nanoformulations

## Abstract

Curcumin, a polyphenol found in turmeric, demonstrates multifaceted anti-cancer activity against glioblastoma. Its therapeutic potential stems from its ability to modulate various molecular pathways implicated in glioblastoma development and progression, enhance the effectiveness of radiation therapy, and induce cancer cell death through diverse mechanisms, including apoptosis, autophagy, and cell cycle arrest. These combined actions make curcumin a promising candidate for glioblastoma treatment, warranting further investigation into its clinical application. In this review, we summarize the latest research on curcumin and its analogs’ potential in glioblastoma therapy.

## 1. Introduction

Glioblastoma (GBM), IDH (isocitrate dehydrogenase) wild-type, WHO (World Health Organization) CNS (Central Nervous System) grade 4, is a rare but highly aggressive brain cancer, representing the most malignant type [1,2,3,4,5]. While its prevalence is relatively low, approximately 10 per 100,000 cases, GBM often leads to fatal outcomes [6].

Glioblastoma symptoms vary based on tumor location, but common manifestations include persistent headaches, seizures, cognitive and personality changes, and neurological deficits like weakness or sensory loss [7,8,9,10]. The aggressive nature of GBM typically results in rapid symptom progression, significantly impacting a patient’s quality of life [7,8]. Early recognition of these clinical signs is crucial for prompt diagnosis and intervention, though the overall prognosis remains poor despite therapeutic advancements, with older adults being more commonly affected [7,9,10].

Diagnosing glioblastoma typically involves a combination of neuroimaging and histopathological examination [3,11,12,13]. Magnetic resonance imaging serves as the primary imaging modality, providing detailed images of the brain to identify and assess the tumor’s extent and distinguish GBM from other brain lesions [11,12]. A definitive diagnosis requires a biopsy, where a tissue sample is microscopically examined to confirm the presence of malignant glial cells [13]. Advanced diagnostic techniques, such as molecular and genetic testing, may also be employed to identify specific markers that can inform treatment strategies and prognostic assessments [12,14,15,16,17].

Glioblastoma’s aggressive nature and resistance to conventional therapies make treatment challenging [18,19,20,21]. The standard approach involves a multimodal strategy encompassing surgical resection, radiation therapy, and chemotherapy [20,21,22,23,24]. Surgery aims to maximize tumor removal while preserving neurological function, but complete resection is often difficult due to the tumor’s infiltrative growth [22,23,25,26,27]. Radiation therapy targets residual tumor cells after surgery to control local growth, while chemotherapy, typically with temozolomide, is administered concurrently to enhance treatment efficacy [21,28,29].

Glioblastoma survival rates have not significantly improved over the past three decades, which underscores the urgent need for exploring more effective novel strategies and therapies [12,14,18,20,21,24,25,27,30,31,32,33,34,35,36,37,38,39].

## 2. Curcumin

Curcumin is a naturally occurring polyphenol extracted from the rhizome of *Curcuma longa*, commonly known as turmeric, a member of the Zingiberaceae (ginger) family [40,41,42]. Widely used as a spice and food colorant, turmeric, and specifically curcumin, has been extensively studied for its diverse pharmacological properties, including anti-inflammatory, antioxidant, and anti-tumor activities [40,41,42,43,44,45,46].

**Figure 1:**
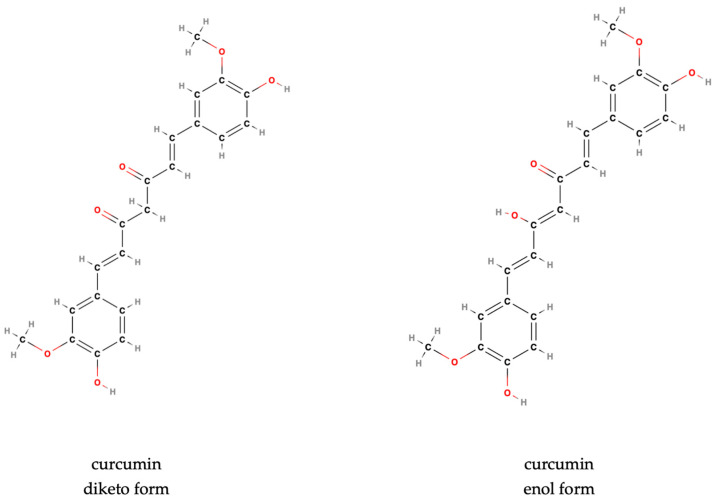
Chemical structure of curcumin—diketo and enol form.

Curcumin, C_21_H_20_O_6_ (Figure 1), formally 1,7-bis(4-hydroxy-3-methoxyphenyl)hepta-1,6-diene-3,5-dione, possesses a β-diketone moiety and methoxyl groups, contributing to its instability and rapid degradation under physiological conditions [47,48]. Curcumin exists in two tautomeric forms (Figure 1): keto-enol and diketo [49,50]. The keto-enol form predominates under alkaline conditions, while the diketo form is more stable in neutral and acidic environments [49,50]. Its lipophilic nature impacts its solubility and absorption in the gastrointestinal tract, limiting bioavailability [48]. Curcumin’s functional groups enable it to act as a Michael acceptor and participate in hydrogen bonding, facilitating interactions with diverse molecular targets [51]. This reactivity underlies its broad biological activities, including antioxidant, anti-inflammatory, and anti-tumor properties [52].

Curcumin undergoes extensive phase I and phase II metabolism, primarily in the liver and intestines, leading to rapid systemic elimination and low bioavailability [51,53,54]. Intestinal microorganisms, such as *Escherichia coli*, metabolize curcumin into dihydrocurcumin and tetrahydrocurcumin through a two-step reduction process, including glucuronidation, sulfation, reduction, and methylation [54,55,56]. The specific formulation of curcumin can substantially alter its metabolism by the gut microbiome, resulting in the generation of unique metabolites that may possess distinct biological properties [54,57]. Moreover, the liver plays a vital role in the metabolism of curcumin, with enzymes such as carboxylesterases and butyrylcholinesterase participating in the hydrolysis of curcumin derivatives [58]. Curcumin has also been shown to interact with cytochrome P450 enzymes, specifically CYP3A4 and CYP2C8, which can influence its pharmacokinetic profile and potential interactions with other pharmaceutical agents [59,60].

### Toxicity

While curcumin exhibits promising therapeutic properties, concerns regarding its toxicity at higher doses have been raised. In vitro studies suggest potential carcinogenic and genotoxic effects, including DNA damage and chromosomal aberrations at low concentrations (10 µg/mL) [61]. Furthermore, curcumin has been shown to impair the function of the tumor suppressor p53 in colon cancer cells [61]. Reproductive toxicity studies have demonstrated that curcumin can adversely affect reproductive health, with significant decreases in sperm motility, capacitation, and fertilization rates observed at concentrations of 5–50 µM [61]. Hepatotoxicity has been observed in clinical cases linked to curcumin intake and animal studies with high dietary turmeric levels (exceeding 30%) [61]. General side effects, such as gastrointestinal disturbances, have been reported at doses ranging from 900 to 3600 mg/day [61]. Acute toxicity studies in mice further indicate toxic symptoms (such as diarrhea, changes in fur and skin, and abnormal walking behavior) at high doses—2000 mg/kg body weight—and significant differences in blood biochemistry and hematology at 1000 mg/kg body weight [62].

A subchronic toxicity study on rats administered turmeric extract at doses of 50, 100, and 200 mg/kg body weight for 28 days revealed no signs of liver or kidney toxicity, suggesting the potential safety of lower doses for long-term use [63]. These findings indicate that while high doses of curcumin may raise toxicity concerns, lower doses could be safe for extended use.

While curcumin may exhibit some toxic effects at high doses, research suggests it offers protection against various other forms of toxicity. Studies have demonstrated curcumin’s ability to mitigate gentamicin-induced renal and cardiac toxicity [64]. This protective effect is attributed to curcumin’s modulation of oxidative stress and inflammatory pathways [64]. Furthermore, curcumin has been shown to alleviate thioacetamide-induced kidney toxicity by enhancing antioxidant systems and reducing oxidative stress and inflammation [65]. Additionally, curcumin has demonstrated efficacy in combating organophosphate pesticide toxicity, highlighting its potential as a protective agent against environmental toxins [66]. These findings suggest that curcumin, despite its potential toxicity at high doses, may offer valuable protective benefits against specific toxins.

Therefore, while curcumin’s therapeutic potential is significant, its safety profile is complex and dose-dependent, warranting further research to fully elucidate its benefit–risk profile.

## 3. Mechanism of Action in Glioblastoma

Curcumin demonstrates multifaceted anti-cancer activities against glioblastoma through various mechanisms (Table 1). It modulates key molecular pathways involved in GBM progression, including those associated with angiogenesis, cell proliferation, and survival. Curcumin’s ability to enhance radiosensitivity further strengthens its potential in GBM therapy, improving the efficacy of radiation treatment. Moreover, curcumin induces cell death through diverse mechanisms, such as apoptosis and autophagy, effectively targeting GBM cells. These combined actions make curcumin a promising therapeutic agent for glioblastoma.

### 3.1. Oxidative Stress

**Figure 2:**
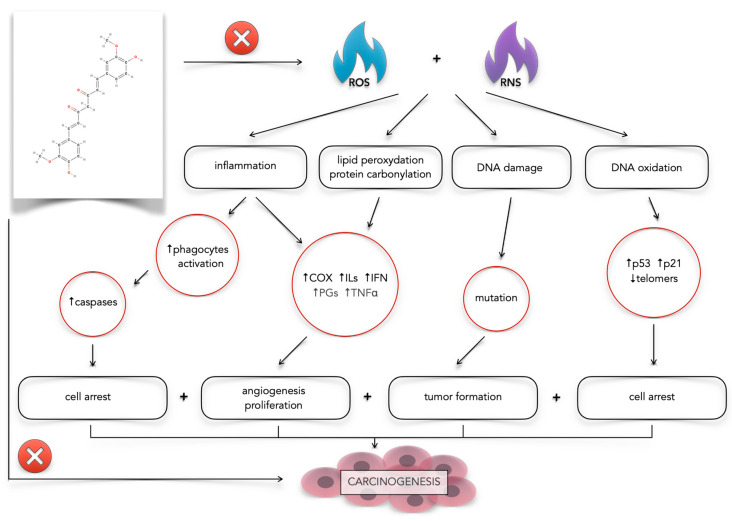
Curcumin and ROS in carcinogenesis: COX—cyclooxygenase; DNA—deoxyribonucleic acid; IFN—interferon; ILs—interleukins; p21—cyclin-dependent kinase inhibitor p21; p53—cellular tumor antigen p53; PGs—prostaglandins; RNS—reactive nitrogen species; ROS—reactive oxygen species; TNF-α—tumor necrosis factor alpha.

Curcumin demonstrates a significant role in modulating oxidative stress within glioblastoma [46,67,68,69,70]. Oxidative stress, an imbalance between reactive oxygen species (ROS) and antioxidants, is implicated in GBM pathogenesis. ROS, being chemically reactive oxygen-containing molecules, play a crucial role in cancer treatment by inducing oxidative stress and damage in cancer cells, ultimately leading to cell death [71]. Glioblastoma cells exhibit elevated reactive oxygen species levels due to impaired production and elimination mechanisms, primarily stemming from mitochondrial dysfunction and inefficient antioxidant systems [71,72,73,74]. While all cells produce ROS from various sources, mitochondria are the primary constitutive source [72]. Further contributing to redox imbalance in GBM are factors like metal ion transitions, peroxisome activity, endoplasmic reticulum stress, and oxidase activity [71,72]. This imbalance in the “oxygen economy” is closely linked to environmental factors influencing tumor growth, differentiation, and survival [74]. Oxidative stress triggers various pathological processes, including the modification of cellular components and biomolecules, leading to genotoxicity [71,75]. Consequently, oxidative stress promotes hypoxia and cellular adaptation, contributing to cancer cell treatment resistance [76]. The enhanced oxidative stress protection observed in cancer stem cells within the tumor mass may also play a role in GBM resistance [73]. Curcumin exhibits antioxidant properties by scavenging ROS and modulating cellular antioxidant defense mechanisms (Figure 2) [46,67,68]. This modulation of oxidative stress contributes to curcumin’s anti-cancer effects in GBM by inhibiting tumor cell proliferation, migration, and invasion and promoting apoptosis [46,68,69,70,77].

Seyithanoglu et al. revealed curcumin’s nuanced role in ROS generation within cancerous and normal cells [78]. This research demonstrates that curcumin’s effect on ROS production is concentration-dependent, with higher curcumin doses leading to increased ROS generation [78]. Importantly, cancer cells (specifically C-6 glioma cells in this study) exhibit greater susceptibility to curcumin-induced ROS production compared to normal cells (L-929 fibroblastic cells), suggesting a degree of selective cytotoxicity [78]. At lower concentrations (10–100 µM), curcumin initially decreases ROS production in both cell types [78]. However, as the concentration increases (20–100 µM), ROS production progressively increases, indicating a non-linear relationship between curcumin concentration and ROS levels [78]. This concentration-dependent effect on ROS production has significant implications for cancer treatment [78]. By selectively increasing ROS in cancer cells, curcumin can induce oxidative stress, leading to DNA damage and ultimately apoptosis [78]. This targeted action holds promise for maximizing therapeutic efficacy while minimizing harm to healthy cells.

Elevated levels of reactive oxygen species can trigger apoptosis, either directly or in conjunction with other cellular processes [79]. In a study by Alkahtani et al., which explored curcumin’s photodynamic efficacy, ROS generation was measured in T98G cells treated with 10 µM curcumin, both with and without blue light exposure (430 nm for 5 and 10 min) [79]. Curcumin treatment alone induced a moderate level of ROS, which was further increased with the duration of blue light exposure [79]. This suggests that blue light enhances curcumin-induced ROS production, contributing to a photodynamic effect and promoting cell death [79]. These findings, based on triplicate experiments, highlight the role of ROS-mediated mechanisms in curcumin’s photodynamic efficacy and its potential in glioblastoma treatment, particularly when combined with blue light.

Other studies show that curcumin reduces cell viability in a dose- and time-dependent manner, with significant inhibition observed at concentrations of 5 µM and above [80]. Morphological changes, such as cells shifting from a flat, elongated shape to a smaller, rounded form, become more pronounced at higher concentrations (above 10 µM), suggesting the induction of apoptosis [80]. Interestingly, curcumin’s effect on calcium signaling appears to be complex [80]. While 5 µM curcumin significantly reduces calcium release, higher doses do not exhibit the same inhibitory effect [80]. Regarding oxidative stress, curcumin exhibits antioxidant properties by decreasing intracellular ROS production [80]. However, it may not provide comprehensive antioxidant support, as evidenced by its lack of effect on GSH-Px (glutathione peroxidase) activity, GSH (glutathione), and lipid peroxidation levels [80]. Curcumin also disrupts mitochondrial function, reducing mitochondrial membrane potential in a concentration-dependent manner, a key event associated with apoptosis [80]. This apoptotic effect is further supported by the observed increase in caspase-3 and -9 activity, particularly at higher curcumin concentrations (e.g., 50 µM) [80]. Overall, these findings suggest that curcumin exerts a protective effect against oxidative stress and modulates calcium release at lower doses, while higher doses induce apoptosis, highlighting its potential as a therapeutic agent for GBM.

The study by Agca demonstrated curcumin’s potent antioxidant effects in U373 glioblastoma cells subjected to high homocysteine (Hcy) levels [81]. Curcumin significantly reduced intracellular reactive oxygen species formation, mitigating oxidative stress, a key contributor to cellular damage [81]. This protective effect stems from curcumin’s activation of the Nrf2 pathway, leading to increased hemoxygenase-1 expression, bolstering the cells’ antioxidant defenses [81]. Furthermore, TP53-induced glycolysis and apoptosis regulator plays a role in curcumin’s antioxidant activity, further reducing Hcy-induced oxidative damage [81]. In essence, curcumin’s antioxidant properties, mediated by Nrf2/HO-1 pathway activation and TIGAR involvement, effectively reduce ROS levels and protect glioblastoma cells from oxidative damage.

Gersey et al. proved that curcumin demonstrates a significant dose-dependent reduction in glioblastoma stem cell (GSCs) viability [82]. Their study showed how curcumin reduces viability in both GSCs and non-stem glioblastoma cells, with an IC50 of approximately 25 μM for GSCs [82]. Even at sub-toxic concentrations (2.5 μM), curcumin effectively inhibits GSC proliferation, sphere formation, and colony-forming potential, hindering the self-renewal crucial for tumor growth and recurrence [82]. Curcumin’s anti-cancer effects are largely mediated by increased reactive oxygen species production within GSCs [82]. This ROS increase, confirmed by fluorescent probes, triggers oxidative stress, damaging cancer cells [82]. Furthermore, curcumin modulates key signaling pathways, activating MAPK (mitogen-activated protein kinase) and inactivating STAT3 (signal transducer and activator of transcription 3), a pathway crucial for cancer cell survival [82]. The antioxidant N-acetylcysteine reverses curcumin’s effects on GSCs, confirming the critical role of ROS induction in curcumin’s mechanism of action [82]. These findings highlight curcumin’s potential as a non-toxic therapeutic strategy against glioblastoma, particularly by targeting GSCs and preventing tumor recurrence.

Curcumin pretreatment of glioblastoma U-87 MG cells prior to exposure to hydrogen peroxide and glucose oxidase-induced oxidative stress enhances cell survival and antioxidant capacity [83]. Curcumin reduces reactive oxygen species levels, with reported decreases of 35% in glucose oxidase-treated cells and 51% in hydrogen peroxide-treated cells [83]. This protective effect is mediated by increased activity and levels of antioxidant enzymes like superoxide dismutase (SOD1 and SOD2) and catalase [83]. Furthermore, curcumin pretreatment reduces the expression of cyclooxygenase-2, an enzyme linked to inflammation, and decreases nitric oxide levels [83]. It also enhances DNA repair mechanisms by increasing the expression of APE1, a crucial DNA repair enzyme [83]. These findings suggest that at non-cytotoxic concentrations, curcumin protects glioblastoma cells against oxidative stress by modulating antioxidant enzymes, reducing inflammation, and promoting DNA repair, highlighting its potential as a supportive treatment strategy.

Temozolomide induces apoptosis in glioblastoma cells by triggering a burst of reactive oxygen species production [84]. Curcumin is also known to stimulate ROS production during apoptosis induced by anti-cancer drugs [84]. When combined, TMZ and curcumin exhibit a synergistic effect, markedly increasing ROS production in U87MG glioblastoma cells beyond the levels observed with either agent alone [84]. This amplified ROS generation contributes to the enhanced therapeutic efficacy of the combination against glioblastoma, sensitizing the cancer cells to TMZ treatment [84]. This synergistic effect on ROS production has been observed in both in vitro and in vivo studies, further supporting the potential of curcumin to augment TMZ’s anti-cancer effects [84].

Luo et al. revealed that curcumin and its analogs, bisdemethoxycurcumin, demethoxycurcumin, and dimethoxycurcumin, increase reactive oxygen species production in glioma cells [85]. This effect is dose-dependent, with higher concentrations leading to greater ROS production [85]. While dimethoxycurcumin showed a less statistically significant increase in ROS in LN229 cells, it still exhibited a trend towards increased ROS, suggesting a potential contribution to ROS-related mechanisms [85]. Further investigation into dimethoxycurcumin’s mechanism of action revealed that it increases ROS production, impacting cellular pathways by reducing p-mTOR and BCL-2 while increasing p-AKT, p-ERK, LC3B-II, and p62, promoting apoptosis and autophagy [85]. The ability of curcumin and its analogs to induce ROS production highlights their potential as therapeutic agents for glioma treatment.

### 3.2. Pl3K/Akt

Curcumin has shown potential in targeting the PI3K/Akt signaling pathway, a critical regulator of cancer cell survival, proliferation, and resistance to apoptosis in glioblastoma [86]. This pathway’s importance in GBM makes it a prime target for therapeutic intervention, and curcumin’s ability to modulate it presents a promising strategy, particularly given GBM’s frequent resistance to conventional treatments. Curcumin exerts its effects by regulating key mediators within the PI3K/Akt pathway, including growth factors and protein kinases [87]. Its inhibitory effect on the PI3K/Akt pathway begins with its suppression of PI3K activation [88]. This action prevents the subsequent phosphorylation and activation of Akt, a crucial protein kinase involved in promoting cell survival and proliferation [88]. By inhibiting Akt activation, curcumin effectively disrupts downstream signaling events that contribute to cancer cell growth and survival [88]. This downregulation of downstream targets leads to a cascade of effects, including cell cycle arrest, the induction of apoptosis, and the inhibition of angiogenesis, ultimately hindering the progression of glioblastoma [87,88].

Curcumin also affects the Akt pathway by increasing the expression of miRs such as miR-223-3p, miR-133a-3p, miR-181a-5p, miR-34a-5p, miR-30c-5p, and miR-1290 [89]. This upregulation impairs the Akt pathway, which is frequently overactive in cancer cells, contributing to enhanced cell survival and resistance to apoptosis [89]. By disrupting Akt signaling, curcumin promotes apoptosis and potentially reduces tumor growth in GBM [89]. This modulation of Akt pathway signaling through microRNA regulation highlights curcumin’s potential as a therapeutic agent in GBM treatment.

Mejía-Rodríguez et al. investigated the effects of combined treatments, including AZD5363, AZD8542, curcumin, and resveratrol, on the PI3K/Akt signaling pathway in human glioblastoma cells [90]. The combinations effectively inhibited the PI3K/Akt pathway, a critical regulator of cell survival and proliferation in cancer cells, leading to a significant decrease in Akt activity [90]. This reduction in Akt activity, a central component of the pathway often overactive in cancers like GBM, effectively disrupted downstream signaling [90]. Consequently, the expression of downstream targets like pP70S6k and pS6k, involved in protein synthesis and cell growth, was reduced [90]. Furthermore, the inhibition of the PI3K/Akt pathway triggered the activation of caspase-3, a marker of apoptosis, indicating that the treatments not only halted cell proliferation but also promoted programmed cell death [90]. These findings highlight the potential of these combined treatments as a promising therapeutic strategy for GBM by effectively targeting the PI3K/Akt pathway and inducing apoptosis.

**Figure 3:**
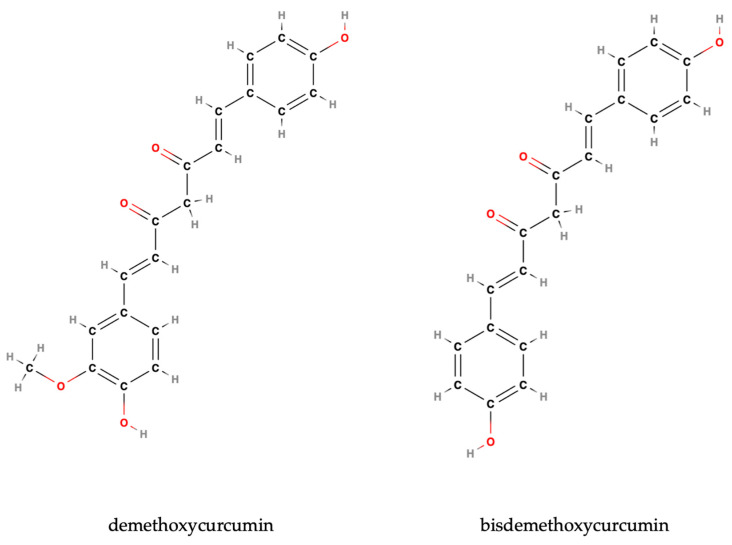
Demethoxycurcumin (DMC) and bisdemethoxycurcumin (BDMC) chemical structure.

Studies on curcumin derivatives—demethoxycurcumin (DMC) and bisdemethoxycurcumin (BDMC) (Figure 3)—showed that they exhibit similar inhibitory effects on the PI3K/Akt pathway in GBM cells [91,92]. They both significantly decrease its activity. A study on demethoxycurcumin and GBM 8401 cells revealed significant anti-cancer properties [91]. DMC effectively inhibited GBM 8401 cell proliferation at concentrations of 1.0–3.0 μM [91]. Further investigations demonstrated DMC’s ability to suppress cell mobility and migration, as evidenced by wound healing and transwell chamber assays [91]. DMC also reduced the activity of MMP-2, a key enzyme in extracellular matrix degradation, suggesting an anti-metastatic effect [91]. Western blot analysis revealed that DMC treatment downregulated several proteins associated with cancer progression, including p-EGFR (active form of epidermal growth factor receptor), GRB2 (growth factor receptor-bound protein 2), PI3K (phosphatidylinositol 3-kinases), p-Akt (phosphorylated Akt), p-PDK1 (phosphorylated 3-phosphoinositide-dependent kinase 1), NF-κB (nuclear factor kappa B), TIMP-1 (tissue inhibitor of metalloprotease-1), MMP-9 (matrix metalloproteinase-9), MMP-2 (matrix metalloproteinase-2), GSK3α/β (glycogen synthase kinase 3α/β), β-catenin, N-cadherin, and vimentin, while upregulating Ras and E-cadherin, proteins associated with reduced metastatic potential [91]. These findings suggest that DMC inhibits cancer cell migration and invasion by suppressing the PI3K/Akt and NF-κB signaling pathways, highlighting its potential as a novel anti-metastasis agent for glioblastoma treatment.

### 3.3. NF-κB

NF-κB, a transcription factor crucial for cell proliferation, survival, and metastasis, is often dysregulated in GBM, making it a prime therapeutic target. Curcumin’s ability to inhibit NF-κB signaling contributes significantly to its anti-cancer effects [93,94]. It is achieved by decreasing the expression of NF-κB-related proteins, resulting in reduced GBM cell proliferation and increased apoptosis [93,95]. Furthermore, curcumin enhances the expression of microRNAs like miR-223-3p and miR-133a-3p, which impair the Akt pathway and inhibit NF-κB, further promoting apoptosis [89].

Bisdemethoxycurcumin (BDMC) exhibits promising anti-metastatic effects in glioblastoma by significantly decreasing NF-κB expression [92]. This reduction in NF-κB, a key regulator of cell migration and invasion, is associated with BDMC’s broader impact on signaling pathways such as PI3K/Akt and Ras/MEK/ERK, which are known to modulate NF-κB activity [92]. By targeting these pathways, BDMC indirectly suppresses NF-κB expression, contributing to its anti-metastatic properties [92]. The downregulation of NF-κB by BDMC is further linked to decreased levels of matrix metalloproteinases (MMP-2 and MMP-9) and N-cadherin, proteins involved in extracellular matrix degradation and cell adhesion, respectively [92]. This reinforces BDMC’s inhibitory effect on GBM cell migration and invasion [92]. These findings highlight BDMC’s potential as a natural compound for developing anti-metastatic therapies in GBM, offering a promising avenue for combating this aggressive cancer.

### 3.4. JAK/STAT

The JAK/STAT (Janus kinase/signal transducer and activator of transcription) signaling pathway plays a crucial role in glioblastoma, driving tumorigenic functions such as proliferation, anti-apoptosis, and immune suppression [96]. Curcumin demonstrates significant anti-tumor effects against glioma by modulating this pathway, both in vitro and in vivo [97]. It achieves this inhibition by suppressing the phosphorylation of JAK1 (Janus kinase 1), JAK2 (Janus kinase 2), and STAT3 (signal transducer and activator of transcription 3), essential components of the pathway [98,99]. Furthermore, curcumin downregulates the transcription of STAT3 target genes, including c-Myc, MMP-9, Snail, and Twist, and the proliferation marker Ki67, thereby reducing cell proliferation, migration, and invasion [98,99]. This downregulation leads to G2/M phase arrest in glioma cells, further suppressing proliferation and inhibiting tumor growth [98,99]. It also reduces the migratory and invasive behavior of glioma cells in a dose-dependent manner, an effect reversible by constitutively active STAT3C, highlighting the role of STAT3 in these processes [98]. In vivo studies using a syngeneic mouse model show that dietary curcumin reduces tumor growth and midline crossing of intracranially implanted tumors, limiting tumor expansion within the brain [98]. This leads to improved long-term survival in mice receiving a curcumin-fortified diet compared to those on a control diet [98]. These findings underscore curcumin’s potential as a safe and promising therapeutic agent for clinical application in glioma therapy.

### 3.5. P53

Curcumin and its analogs (like PGV-1—pentagamavunone-1, and CCA-1.1—chemoprevention-curcumin analog-1.1) demonstrate promising anti-cancer effects in glioblastoma by modulating the p53 pathway, a critical regulator of cell cycle and apoptosis [100]. It increases p53 expression in GBM cells, promoting apoptosis and inhibiting cell proliferation [100]. This is achieved by suppressing the p-AKT/mTOR pathway and enhancing PTEN expression, both crucial in tumor growth and survival [101]. Beyond increasing wild-type p53 (WTp53) expression, curcumin exhibits a unique effect on mutant p53 (Mutp53) in cancer cells, leading to its ubiquitination and destabilization without affecting wild-type p53 [102]. This destabilization is not reversed by proteasome and lysosome inhibitors, suggesting a mechanism independent of these pathways [102]. Curcumin treatment also causes reversible nuclear aggregation of Mutp53, potentially linked to oxidative stress or disulfide bond formation, as treatment with Dithiothreitol reverses the aggregation [102]. A broad-spectrum deubiquitinase inhibitor induces similar Mutp53 aggregation, implying that curcumin may act by inhibiting deubiquitinases [102]. Importantly, it selectively inhibits the colony-forming abilities of Mutp53-expressing cells, inducing cytoplasmic vacuolation and cell death specifically in these cells [102]. This selective cytotoxicity highlights curcumin’s potential as a targeted therapeutic agent against Mutp53-expressing cancers [102]. These findings collectively suggest curcumin’s promise as a therapeutic agent, specifically targeting Mutp53 while sparing WTp53, offering potential specificity and efficacy in cancer therapy.

Curcumin also modulates several signaling pathways, including the p53-BCL2 network, involved in cell death processes like paraptosis [103]. This modulation occurs through changes in microRNA expression and ER stress response genes, further supporting curcumin’s role in inducing cell death [103]. Moreover, it downregulates several microRNAs, including miR-27a-5p, miR-221-3p, miR-21-5p, miR-125b-5p, and miR-151-3p, leading to the inhibition of the p53-BCL2 pathway, a key regulator of apoptosis [89]. Specifically, by inhibiting miR-21 expression, curcumin activates apoptosis through the activation of caspase-3 and death receptors 4 and 5, amplifying the apoptotic response in GBM cells [89]. Additionally, curcumin’s reduction in miR-27a expression enhances the expression of C/EBP homologous protein, inducing paraptosis, another form of programmed cell death, further contributing to its anti-tumor effects [89]. These findings highlight curcumin’s multifaceted approach to inducing cell death in GBM by modulating the p53 pathway through microRNA regulation, suggesting its potential as a therapeutic agent in cancer treatment.

### 3.6. MAPK

The mitogen-activated protein kinase (MAPK) pathway plays a critical role in regulating cell proliferation, differentiation, and apoptosis, making it a key target in cancer therapy. Curcumin inhibits the ERK/MAPK pathway, which is activated in the stress-induced proliferation and invasion of glioma cells [104]. This inhibition leads to a decreased expression of matrix metalloproteinases (MMP-2/9) and CD147, proteins associated with tumor invasion and metastasis [104]. Additionally, curcumin suppresses the phosphorylation of ERK1/2, a crucial component of the MAPK pathway, further reducing GBM cell proliferation and invasion [104].

Su et al. showed that treatment with demethoxycurcumin decreased the levels of key MAPK pathway proteins, including p-Raf, MEK, and p-ERK1/2, in GBM 8401 cells [91]. This downregulation of the MAPK pathway contributes to DMC’s ability to suppress proliferation, migration, and invasion of these cells [91]. The inhibition of the MAPK pathway complements DMC’s effects on other critical cancer-related pathways, such as PI3K/Akt and NF-κB, further enhancing its anti-cancer potential [91].

Bisdemethoxycurcumin demonstrates promising anti-neoplastic activity in glioblastoma models by inhibiting the Ras/MEK/ERK pathway, a critical component of the MAPK signaling cascade [92]. This pathway is essential for cell proliferation and survival, and its downregulation is linked to reduced cancer aggressiveness [92]. BDMC’s inhibitory action on this pathway leads to the suppression of cell proliferation and metastasis in GBM cells [92]. The mechanism involves a decreased expression of downstream proteins like NF-κB, which subsequently reduces the levels of MMP-2 and MMP-9, enzymes crucial for cell migration and invasion [92]. By disrupting the signaling required for GBM cell migration and invasion, BDMC acts as an effective anti-metastatic agent.

### 3.7. Shh

Curcumin’s interaction with the Sonic Hedgehog (Shh) pathway, a critical regulator of cell proliferation and differentiation often dysregulated in glioblastoma, highlights its potential as a therapeutic agent. Curcumin inhibits the Shh/GLI1 signaling pathway, leading to decreased expression of GLI1 target genes like CyclinD1, Bcl-2, and Foxm1, which are involved in cell cycle regulation and apoptosis resistance [105]. This inhibitory effect is amplified when curcumin is combined with miR-326, resulting in enhanced cytotoxicity and apoptosis in glioma cells while concurrently reducing proliferation and migration, regardless of p53 status [106]. The combination of miR-326 and curcumin leads to a substantial inhibition of the Shh/GLI1 signaling pathway [106]. This inhibitory effect is markedly stronger than that observed with either treatment alone and is independent of p53 status, broadening its potential therapeutic scope [106]. In vivo studies further corroborate these findings, demonstrating reduced tumor volume and improved survival in animal models treated with the combination therapy compared to monotherapy [106]. This suggests that miR-326 alters the anti-glioma mechanism of curcumin, enhancing its overall therapeutic efficacy [106]. Therefore, the combination of miR-326 and curcumin presents a promising strategy for glioblastoma treatment by increasing glioma cell chemosensitivity to curcumin, primarily through SHH/GLI1 pathway inhibition.

Du et al. studied curcumin’s anti-cancer effects in glioblastoma through the downregulation of the Shh/GLI1 signaling pathway and GLI1 (glioma-associated oncogene homolog 1) target genes expression [105]. This downregulation occurs at both the mRNA and protein levels, affecting key components of the pathway such as Shh, Smo, and GLI1 [105]. The inhibitory effect is dose- and time-dependent, suggesting that higher curcumin concentrations and longer exposure durations enhance its impact on the pathway [105]. Furthermore, curcumin impacts downstream targets of GLI1, downregulating the expression of GLI1-dependent genes like CyclinD1, Bcl-2, and Foxm1, which are crucial for cell proliferation and survival [105]. Curcumin also inhibits the nuclear translocation of GLI1, a critical step for its transcriptional activity and role in cancer cell proliferation [105]. This multifaceted inhibition of the SHH/GLI1 pathway translates into tangible anti-cancer effects, including suppressed cell proliferation, colony formation, and migration, and increased apoptosis, partially mediated through the mitochondrial pathway, as evidenced by an increased Bax/Bcl2 ratio [105]. In vivo studies using a U87-implanted nude mice model further support these findings, demonstrating reduced tumor volume, decreased GLI1 expression, and prolonged survival following curcumin treatment [105].

Research by Mejía-Rodríguez et al. showed that curcumin, in combination therapies, effectively inhibits the Shh pathway, leading to a reduction in tumor growth and survival [90]. This inhibition is marked by a decrease in the expression of key SHH pathway components, including SMO and GLI1, essential proteins for SHH signaling activation and propagation [90]. The reduced expression of these components suggests that curcumin disrupts the SHH pathway, potentially limiting the aggressive nature of GBM [90]. The combination of curcumin with compounds like AZD5363, AZD8542, and resveratrol exhibits synergistic effects, suppressing both the PI3K/AKT and SHH pathways, targeting multiple survival mechanisms in cancer cells and potentially enhancing cell death while reducing treatment resistance [90]. Furthermore, the inhibition of the SHH pathway by curcumin-containing treatments is linked to caspase-3 activation, a marker of apoptosis, indicating that curcumin not only disrupts survival signaling but also actively promotes programmed cell death, a desirable outcome in cancer therapy [90]. These findings highlight the potential of curcumin, especially in combination therapies, as a valuable therapeutic agent against GBM by targeting the SHH signaling pathway.

### 3.8. Radiosensitization

Curcumin has emerged as a promising radiosensitizer in glioblastoma treatment. Studies exploring the combination of curcumin and radiotherapy have demonstrated enhanced therapeutic efficacy through various mechanisms, including increased apoptosis, cell cycle arrest, and the modulation of stress signaling pathways. These effects contribute to improved outcomes in glioblastoma models. Importantly, curcumin exhibits a radioprotective effect on normal cells while simultaneously sensitizing cancer cells to radiation, suggesting a potential for improved therapeutic efficacy with reduced side effects [107].

Curcumin’s radiosensitizing effect primarily stems from its ability to modulate key molecular pathways, notably the mammalian target of rapamycin (mTOR), which plays a crucial role in autophagy induction [108]. By activating autophagy, curcumin increases cancer cells’ susceptibility to radiation-induced damage, thereby enhancing treatment efficacy [108]. Furthermore, curcumin targets glioblastoma stem cells, a highly tumorigenic population often responsible for therapeutic resistance and tumor recurrence [108]. By suppressing GSCs’ tumorigenic properties through autophagy induction, curcumin not only enhances radiosensitivity but also helps reduce the risk of relapse [108].

Moreover, curcumin promotes immunogenic cell death (ICD) by activating endoplasmic reticulum stress pathways, leading to increased apoptosis and immune response, as evidenced by elevated calreticulin exposure and release of HSP70 (70 kilodalton heat shock proteins) and ATP (adenosine triphosphate). A study investigating the combined effects of curcumin and ionizing radiation in glioma cells revealed promising results [109]. The combination therapy significantly increased apoptosis in glioma cells under both normoxic and hypoxic conditions [109]. Furthermore, curcumin enhanced immunogenic cell death by increasing the exposure of calreticulin on the cell surface and promoting the release of HSP70 and ATP, key markers of ICD and indicators of an enhanced immune response against tumor cells [109]. This study demonstrated that curcumin activates ER stress signaling pathways, specifically the PERK-eIF2α and IRE1α-XBP1 pathways, which play a crucial role in the observed increase in ICD [109]. Inhibiting these pathways diminished the effects of curcumin and X-ray on apoptosis and CRT exposure, highlighting their importance in the process [109]. In vivo experiments using mouse models further supported these findings, showing that the combination of curcumin and X-ray irradiation elicited a stronger immune response compared to radiation alone, evidenced by a higher tumor rejection rate (90% vs. 70%) [109]. This enhanced immune response was accompanied by an increased infiltration of CD4+ and CD8+ T lymphocytes and CD11c+ dendritic cells into the tumor microenvironment, further demonstrating curcumin’s potential to augment the efficacy of radiotherapy in glioblastoma treatment [109].

Curcumin amplifies reactive oxygen species generation, enhancing radiation’s cytotoxic effects and resulting in increased apoptosis and reduced cell invasion. Studies have shown that curcumin enhances the efficacy of radiation therapy under both high and low Linear Energy Transfer conditions in vitro [77]. When combined with radiation, curcumin increases the sub-G1 cell population, elevates reactive oxygen species levels, and ultimately leads to increased apoptosis in glioblastoma cells [77]. These radiosensitizing effects are more pronounced with neutron (high LET) radiation compared to γ (low LET) radiation [77]. Furthermore, the combination of curcumin and neutron radiation significantly inhibits glioblastoma cell invasion, exceeding the effects of either treatment alone or curcumin combined with γ-ray treatment [77]. This enhanced radiosensitivity, particularly with high LET radiation, suggests curcumin’s potential clinical utility as part of a combination therapy strategy to improve outcomes for glioblastoma patients.

The combination of curcumin and radiation also induces cell cycle arrest at the G2/M phase, further inhibiting glioblastoma cell proliferation. Zoi et al. investigated the potential of curcumin as a radiosensitizer for glioblastoma [110]. Using U87 and T98 glioblastoma cell lines, researchers pretreated cells with curcumin before exposing them to radiation doses of 2 Gy or 4 Gy [110]. The combination treatment’s effects were compared to those of curcumin or radiation alone, assessing cell viability and proliferation with trypan blue exclusion and MTT assays, respectively [110]. Synergistic effects were analyzed using CompuSyn software, and cell cycle progression was examined via flow cytometry [110]. The results showed that the combination of curcumin and radiation significantly reduced cell viability in both cell lines compared to either treatment alone [110]. Moreover, the combination treatment induced a more pronounced G2/M cell cycle arrest than either treatment alone [110]. Interestingly, combining curcumin with temozolomide also resulted in increased tumor cell death [110]. This study concluded that low-dose curcumin combined with irradiation exhibits a strong synergistic anti-proliferative effect on glioblastoma cells in vitro, suggesting a promising new therapeutic strategy; however, further research is needed to elucidate the underlying molecular mechanisms [110].

Ghanbari et al. explored the combined effects of curcumin, radiation therapy, and hyperthermia on a glioblastoma spheroid model [111]. Curcumin, known for its anti-inflammatory and antioxidant properties, was hypothesized to enhance the effects of radiation and hyperthermia, potentially leading to better treatment outcomes [111]. The results demonstrated a significant impact of the combined treatment on the glioblastoma spheroid model, suggesting that curcumin may sensitize tumor cells to radiation and heat, thereby enhancing overall treatment efficacy [111].

Wang et al. utilized a rat model of glioblastoma to investigate the potential of curcumin as a radiosensitizer [112]. Researchers developed an orthotopic F98/FGT glioma-bearing rat model using lentivirus transduction of triple-reporter genes (Fluc/GFP/tk) into F98 glioblastoma cells [112]. This model allowed for noninvasive monitoring of tumor growth and treatment response [112]. This study evaluated the therapeutic efficacy of curcumin alone, radiation alone, and a combination of the two using bioluminescent imaging and overall survival measurements [112]. Curcumin induced G2/M cell cycle arrest in F98 cells, sensitizing them to radiation [112]. In the animal model, the combination of curcumin and radiotherapy synergistically suppressed the growth of both transplanted and in situ brain tumors, leading to significantly extended survival periods compared to either treatment alone [112]. These findings suggest that curcumin may act as a novel radiosensitizer, enhancing the therapeutic efficacy of radiotherapy in glioblastoma. The triple-reporter animal model proved valuable for evaluating therapeutic efficacy and provides a promising approach for future research and potential clinical applications.

On the other hand, a study by Sminia et al., investigating curcumin’s potential as a radiosensitizer in glioblastoma using U251 human glioma cells, came to slightly different conclusions [113]. The researchers sought to determine the optimal curcumin dose and exposure duration for radiosensitization [113]. Experiments using curcumin concentrations up to 100 μM and exposure times from 0.5 to 96 h revealed that 96 h exposure to 5 μM curcumin inhibited U251 cell proliferation without significantly affecting cell survival [113]. Higher curcumin doses (greater than 5 μM for 96 h, beyond 25 μM for 2 h, and 100 μM for more than 0.5 h) reduced U251 cell survival, but these effects were absent at the lower 5 μM dose used in combination with radiation [113]. Combining a 72 h, 5 μM curcumin exposure with single-dose or fractionated radiation (five daily fractions of 2 Gy) showed no interaction between curcumin and radiation, indicating no radiosensitizing effect [113]. Clonogenic cell survival curves confirmed the absence of radiosensitization [113]. This study concluded that curcumin does not exhibit a radiosensitizing effect at clinically achievable concentrations in GBM treatment—as reported, intratumoral curcumin concentrations are too low for cytotoxic effects or synergistic interaction with radiation [113].

**Table 1:** Curcumin’s glioblastoma targets.

Target	Treatment	References
Oxidative stress	curcumin	[78,80,82,83,85]
	curcumin + blue light	[79]
	curcumin + homocysteine	[81]
	curcumin + temozolomide	[84]
	bisdemethoxycurcumin	[85]
	demethoxycurcumin	[85]
	dimethoxycurcumin	[85]
Pl3K/Akt	curcumin	[88,89]
	curcumin + AZD5363, AZD8542, resveratrol	[90]
	bisdemethoxycurcumin (BDMC)	[92]
	demethoxycurcumin (DMC)	[91]
NF-κB	bisdemethoxycurcumin (BDMC)	[92]
JAK/STAT	curcumin	[98]
p53	curcumin	[100,101,102,103]
	PGV-1 (pentagamavunone-1)	[100]
	CCA-1.1 (chemoprevention-curcumin analog-1.1)	[100]
MAPK	curcumin	[104]
	bisdemethoxycurcumin (BDMC)	[92]
	demethoxycurcumin (DMC)	[91]
Shh	curcumin	[105,106]
	curcumin + AZD5363, AZD8542, resveratrol	[90]
Radiosensitization	curcumin	[77,108,109,110,111,112,113]

## 4. Curcumin vs. Analogs

Curcumin analogs have emerged as promising therapeutic agents for glioblastoma, demonstrating superior anti-tumor activity at lower concentrations compared to curcumin itself. This enhanced efficacy is attributed to their improved solubility and stability and their ability to induce cell cycle arrest and apoptosis in GBM cells. Importantly, these analogs exhibit minimal effects on normal cells, further enhancing their potential as targeted GBM therapies.

CCA-1.1 demonstrates improved solubility and stability compared to curcumin, making it a more promising candidate for glioblastoma treatment [100]. Studies have shown that CCA-1.1 exhibits higher cytotoxicity against GBM cells than curcumin, evidenced by a lower IC50 value of 9.8 μM, significantly lower than the 40 μM IC50 of temozolomide, a standard GBM treatment [100]. Furthermore, CCA-1.1 effectively targets the epidermal growth factor receptor, often mutated in GBM, demonstrating stronger binding and inhibition of mutant EGFR compared to curcumin [100]. This targeted action, combined with its potential to modulate the immune environment, positions CCA-1.1 as a promising therapeutic agent for GBM, warranting further clinical investigation.

The study by Razali et al. investigated the effects of curcumin analogs, FLDP-5 and FLDP-8, designed for improved bioavailability and potency, on LN-18 human glioblastoma cells [114]. Both analogs exhibited significantly higher cytotoxicity compared to curcumin, with lower IC50 values of 2.5 µM and 4 µM for FLDP-5 and FLDP-8, respectively, compared to 31 µM for curcumin [114]. The enhanced potency of the analogs was linked to increased oxidative stress in LN-18 cells, marked by elevated levels of reactive oxygen species [115]. Furthermore, FLDP-5 and FLDP-8 demonstrated anti-migratory effects, inhibiting both migration and invasion of LN-18 cells in a dose-dependent manner [114]. Interestingly, the analogs induced S-phase cell cycle arrest, distinct from curcumin’s G2/M phase arrest [114]. DNA damage, linked to increased ROS production, was also more pronounced in cells treated with the analogs [114]. Importantly, predictions suggest both analogs are capable of crossing the blood–brain barrier, a crucial factor for effective GBM treatment, with FLDP-5 showing a particularly high probability [114]. These findings highlight the potential of FLDP-5 and FLDP-8 as promising candidates for further research and development in glioblastoma treatment.

Inai et al. investigated the anti-tumor activity of curcumin analogs, Compound A (ComA) and Compound B (ComB), against temozolomide-resistant glioblastoma cell lines, U87-MG and U251 [115,116]. Using the MTT assay to assess cell viability, the researchers determined IC50 values, representing the concentration needed to inhibit 50% of cell growth [115,116]. For U87-MG cells, the IC50 values were 9.78 µM for curcumin, 2.42 µM for ComA, and 1.28 µM for ComB [115,116]. For U251 cells, the IC50 values were 9.50 µM for curcumin, 2.27 µM for ComA, and 0.64 µM for ComB [115,116]. These results demonstrate the increased efficacy of ComA and ComB compared to curcumin, requiring lower concentrations to achieve similar anti-tumor effects [115,116]. Importantly, neither ComA nor ComB reduced cell viability in primary cultured astrocytes from neonatal rats at concentrations effective against GBM cells, indicating selective toxicity towards cancer cells [115,116]. Mechanistically, both ComA and ComB induced G2/M phase arrest and apoptosis in GBM cells, accompanied by a decrease in mRNA expression levels of cell cycle-related proteins CDK1 and CyclinB1 [115,116]. These findings indicate that both ComA and ComB exhibit enhanced anti-tumor activity against glioblastoma cells compared to curcumin, requiring lower concentrations to achieve similar effects [115,116]. Importantly, neither ComA nor ComB reduced the viability of primary cultured astrocytes from neonatal rats at concentrations effective against GBM cells, suggesting selective cytotoxicity towards cancer cells [115,116]. Mechanistically, both ComA and ComB induced cell cycle arrest at the G2/M phase and triggered apoptosis in GBM cells, accompanied by a decrease in the expression of cell cycle-related proteins CDK1 and CyclinB1 [115,116].

Luo et al. compared the anti-cancer activity of curcumin and its analogs, dimethoxycurcumin, demethoxycurcumin, and bisdemethoxycurcumin, in glioma cells [85]. While dimethoxycurcumin showed selective cytotoxicity, being more toxic to glioma cells than normal brain cells, curcumin exhibited the highest overall cytotoxicity among the tested compounds [85]. All four compounds induced cell cycle arrest, increasing the sub-G1 and G2/M phases while decreasing the G1 phase [85]. They also increased apoptosis in a dose-dependent manner, as evidenced by increased annexin-V staining [85]. However, their effects on proliferation varied; curcumin suppressed proliferation, demethoxycurcumin’s effect was cell line-dependent, dimethoxycurcumin had minimal impact, and bisdemethoxycurcumin actually increased proliferation [85]. Curcumin and dimethoxycurcumin inhibited migration and colony formation, although dimethoxycurcumin’s effect was only observed at high doses, while bisdemethoxycurcumin promoted colony formation [85]. All compounds increased ROS production [85]. Dimethoxycurcumin’s mechanism involved modulating p-mTOR, BCL-2, p-AKT, p-ERK, LC3B-II, and p62, suggesting a complex interplay between autophagy and apoptosis [85]. Overall, curcumin demonstrated the strongest anti-cancer activity, followed by dimethoxycurcumin, demethoxycurcumin, and lastly, bisdemethoxycurcumin.

## 5. Novel Delivery Systems

Curcumin holds promise in glioblastoma treatment due to its antineoplastic properties; however, its clinical application is hampered by poor bioavailability and limited ability to cross the blood–brain barrier. To address these challenges, researchers have developed novel delivery systems aimed at improving curcumin’s solubility, stability, and targeted delivery to tumor cells (Table 2) [117,118,119,120,121]. These advancements seek to enhance curcumin’s therapeutic efficacy against GBM, potentially establishing it as a viable treatment option for this aggressive cancer.

A study by Keshavarz et al. investigating the combined effects of dendrosomal nanocurcumin (DNC) and p53 overexpression in glioblastoma cells revealed promising results [122]. MTT assays demonstrated that DNC effectively inhibits U87-MG cell proliferation in a time- and dose-dependent manner, indicating that higher DNC concentrations and longer exposures lead to greater reductions in cell viability [122]. Furthermore, combining p53 overexpression with DNC treatment significantly increased apoptosis, with 90% of cells undergoing apoptosis compared to 15% with p53 overexpression alone and 38% with DNC alone, suggesting a synergistic effect [122]. Real-time PCR analysis revealed that the combination treatment enhanced the expression of GADD45, a gene associated with DNA damage response and cell cycle arrest, while reducing the expression of NF-κB and c-Myc, both involved in promoting cancer cell survival and proliferation [122]. These findings suggest that combining DNC with p53 overexpression could be a promising therapeutic strategy for glioblastoma, offering a novel and efficient approach by merging gene and drug delivery systems.

Tondro et al. examined the anti-inflammatory effects of nanocurcumin versus free curcumin on U87 glioblastoma cells by measuring the secretion of pro-inflammatory cytokines IL6 (interleukin 6) and TNF-α (tumor necrosis factor-α) [123]. U87 cells were treated with 84.87 µg/mL of nanocurcumin and 47 µg/mL of free curcumin, and cytokine production was assessed using ELISA (enzyme-linked immunosorbent assay) [123]. Both nanocurcumin and free curcumin significantly reduced IL6 and TNF-α secretion [123]. However, nanocurcumin demonstrated superior efficacy in inhibiting cytokine production compared to free curcumin [123]. These findings suggest that nanocurcumin possesses enhanced anti-inflammatory properties in glioblastoma cells, highlighting its potential as a therapeutic agent for mitigating glioblastoma-associated inflammation.

Hesari et al. found that nanomicelle curcumin significantly impacts the NF-κB pathway in GBM cells by decreasing the expression of p65, a crucial subunit of the NF-κB complex [95]. This downregulation of NF-κB leads to decreased tumor cell proliferation and increased apoptosis [95]. While erlotinib, an EGFR tyrosine kinase inhibitor, acts through a different mechanism, curcumin’s modulation of NF-κB offers a complementary approach, potentially overcoming the limitations of current GBM therapies [95]. These findings suggest that nanomicelle curcumin holds promise as a therapeutic agent for GBM due to its targeted action on the NF-κB pathway.

Bagherian et al. studied the effects of curcumin, nanomicellar-curcumin, and temozolomide, alone and in combination, on U87 glioblastoma cells [124]. All treatments, except for 20 μM curcumin alone, significantly reduced cell viability [124]. Curcumin at 50 μM, nanomicellar-curcumin, and the combination of nanomicellar-curcumin and TMZ significantly inhibited cell invasion and migration [124]. The treatments increased the levels of autophagy-related proteins (Beclin 1, LC3-I, and LC3-II), suggesting the induction of autophagy [124]. Apoptosis was also promoted, as evidenced by increased Bcl-2 and caspase 8 levels and decreased Bax levels [124]. Furthermore, the treatments significantly downregulated genes associated with the Wnt signaling pathway (β-catenin, cyclin D1, Twist, and ZEB1), a pathway implicated in GBM progression and drug resistance [124]. These findings highlight the potential of nanomicellar-curcumin and TMZ combination therapy as a promising strategy for treating glioblastoma.

The study by He et al. investigated a novel targeted drug delivery system using curcumin/Fa-PEG-PLA (curcumin/polylactic acid-polyethylene glycol-folate) nanoparticles for enhanced glioblastoma treatment [125]. They demonstrated superior efficacy in suppressing GL261 cell growth compared to free curcumin and Cur/MPEG-PLA (curcumin/methoxy poly (ethylene glycol)-poly (L-lactic acid)), indicating enhanced apoptosis induction [125]. In vivo studies using tumor-bearing mice showed that the curcumin/Fa-PEG-PLA complex effectively repressed tumor growth in both subcutaneous and intracranial models by suppressing angiogenesis and promoting apoptosis [125].

Liang et al. developed a nanogel by co-loading curcumin (Cur) and temozolomide (TMZ) into PEG-PLGA (polyethylene glycol-poly(lactic-co-glycolic acid)) nanoparticles, which were then encapsulated within a thermosensitive hydrogel [126]. This injectable nanogel was designed for post-surgical application in the glioblastoma resection cavity [126]. The nanogel exhibited an excellent drug-loading capacity and sustained drug release, ensuring prolonged therapeutic drug levels at the target site [126]. Importantly, the nanogel effectively inhibited the recurrence of TMZ-resistant tumors, addressing a major challenge in glioblastoma treatment [126]. Furthermore, the nanogel demonstrated low drug-induced toxicity, suggesting a safer alternative to conventional chemotherapy [126]. The maintained Cur/TMZ ratio throughout the study ensured consistent synergistic effects of the combined drugs [126]. Overall, Cur/TMZ nanogel holds promise for the localized inhibition of GBM recurrence, offering a targeted therapeutic approach with the potential to improve patient outcomes.

Ghoreyshi et al. investigated the effects of curcumin nanoparticles (CU-NPs) on glioblastoma cells [67]. The CU-NPs were characterized by a size of 77.27 nm, a polydispersity index of 0.29, and a zeta potential of −22.45 mV, indicating suitable size, uniform distribution, and moderate stability for cellular uptake [67]. With a high encapsulation efficiency of approximately 98%, the nanoparticles effectively incorporated curcumin [67]. Treatment with CU-NPs led to a decrease in intracellular reactive oxygen species and malondialdehyde levels, suggesting a reduction in oxidative stress [67]. Furthermore, CU-NP treatment increased both the gene expression and activity of key antioxidant enzymes, including superoxide dismutase, catalase, glutaredoxin, and thioredoxin, enhancing the cellular antioxidant defense system [67]. These findings suggest that CU-NPs may offer a promising approach to glioblastoma treatment by modulating the antioxidant–oxidant balance, reducing oxidative stress, and bolstering antioxidant defenses within cancer cells.

A study by Gallien et al. investigated the efficacy of curcumin encapsulated in surface-modified polyamidoamine dendrimers in reducing the viability of glioblastoma cell lines [127]. Three variations in PAMAM dendrimers were tested: a traditional PAMAM dendrimer with a 100% amine surface (G4 NH2), a surface-modified dendrimer with 10% amine and 90% hydroxyl groups (G4 90/10-Cys), and the latter loaded with curcumin (G4 90/10-Cys-Cur) [127]. This study utilized mouse, rat (F98), and human (U87) glioblastoma cell lines [127]. Cell viability was assessed using the MTT assay, a standard method for measuring cellular metabolic activity [127]. The results showed that the curcumin-loaded dendrimer (G4 90/10-Cys-Cur) significantly reduced the viability of all three glioblastoma cell lines compared to non-cancerous control cells [127]. Importantly, unencapsulated curcumin did not show similar efficacy, highlighting the importance of the dendrimer encapsulation for enhanced delivery and therapeutic effect [127]. The traditional PAMAM dendrimer (G4 NH2) exhibited significant toxicity to both cancerous and normal cells, indicating a lack of specificity and potential for adverse effects [127]. These findings suggest that curcumin, when delivered via surface-modified dendrimers, offers a promising therapeutic strategy for glioblastoma by selectively targeting cancer cells while sparing healthy cells. This underscores the potential of using nanotechnology to optimize the delivery and efficacy of antioxidants like curcumin in cancer treatment.

Hou et al. worked on curcumin-loaded poloxamer188-based nanoparticles (P188TT NPs) for glioma treatment [128]. The successful synthesis of the P188TT copolymer was confirmed using 1H NMR (Proton Nuclear Magnetic Resonance), Raman, and FITC (Fluorescein Isothiocyanate) spectroscopy [128]. The P188TT NPs demonstrated a low critical micelle concentration, indicating their stability and suitability for drug delivery [128]. Characterization and bio-safety assessments revealed appropriate size, zeta potential, good stability, and ideal bio-safety profiles [128]. Curcumin-loaded P188TT NPs (Cur/P188TT NPs) were analyzed using DSC (differential scanning calorimetry) and TGA (Thermogravimetric Analysis) to determine their thermal properties and stability [128]. In vitro release studies showed a faster curcumin release rate at pH 6.8 compared to pH 7.4, suggesting enhanced efficacy in the slightly acidic tumor microenvironment [128]. Importantly, the nanoparticles exhibited good brain-targeting efficiency, which is crucial for glioma treatment [128]. Cellular uptake assays demonstrated enhanced curcumin uptake in glioma cells, and MTT tests confirmed increased anti-tumor activity of the encapsulated drug [128]. These findings highlight the potential of Cur/P188TT NPs as a promising therapeutic strategy for glioma.

In the study by Schulze et al., curcumin-loaded lipid nanoparticles were prepared using dual asymmetric centrifugation and characterized via dynamic light scattering, laser Doppler velocimetry, and atomic force microscopy [129]. The nanoparticles’ photodynamic efficacy against glioblastoma was evaluated using the chorioallantois membrane model, chosen for its three-dimensional nature and extensive vascularization [129]. Xenografted U87 glioblastoma cells on the CAM (chorioallantois membrane) were treated topically, targeting both the tumor and surrounding vasculature [129]. The effects of photodynamic therapy with curcumin-loaded nanoparticles were assessed through microscopic examination, imaging techniques (positron emission tomography and X-ray computed tomography), and histological analysis of excised xenografts using hematoxylin and eosin and KI67 staining [129]. These methods allowed for a comprehensive evaluation of the treatment’s impact on tumor viability, growth, and angiogenesis [129]. The results suggest that curcumin-loaded lipid nanoparticles may enhance photodynamic therapy for glioblastoma, offering a promising direction for future cancer treatment research.

Negah et al. developed curcumin-loaded niosome nanoparticles (CM-NPs) characterized by a diameter of approximately 60 nm and a zeta potential of around −35 mV, indicating stable physicochemical properties suitable for drug delivery [130]. These CM-NPs demonstrated significantly enhanced anti-tumor effects against glioblastoma stem-like cells compared to free curcumin [130]. The enhanced efficacy was evident in the significant reduction in cell viability, proliferation, and migration of GSCs (glioblastoma stem-like cells) [130]. Furthermore, CM-NPs induced higher levels of apoptosis and cell cycle arrest in GSCs compared to free curcumin, accompanied by increased expression of the pro-apoptotic marker Bax and decreased expression of the anti-apoptotic marker Bcl2 [130]. The enhanced anti-tumor activity of CM-NPs was also linked to a significant increase in reactive oxygen species production in GSCs [130]. CM-NPs effectively impaired GSC migration and invasiveness, potentially through MCP-1-mediated pathways, and reduced the secretion of MMP-2, a protein involved in cancer cell migration and invasion [130]. Similar to free curcumin, CM-NPs also inhibited colony formation and reduced the expression of cancer stem cell markers like Sox2 and nestin [130]. These findings suggest that CM-NPs may be an ideal delivery system for curcumin in glioblastoma treatment, offering a promising therapeutic approach. However, further in vivo studies are needed to validate these findings.

Tondro et al. investigated the anti-tumor effects of curcumin-loaded niosome nanoparticles (CM-NPs) compared to free curcumin (CM) on U87 MG (Uppsala 87 Malignant Glioma) glioblastoma cells [131]. Both CM and CM-NPs reduced cell proliferation in a time- and dose-dependent manner, but CM-NPs induced significantly higher levels of apoptosis [131]. CM-NPs also exhibited superior inhibition of cell migration, demonstrated through a wound healing assay [131]. Furthermore, CM-NP treatment resulted in a significant increase in reactive oxygen species production, suggesting a potential mechanism for its enhanced anti-tumor activity [131]. CM-NPs effectively downregulated the expression of tumor progression markers like NF-κB and STAT3 at the mRNA level and reduced the production of pro-inflammatory cytokines IL-1β and TGF-β [131]. Increased DNA fragmentation in U87 cells treated with CM-NPs further confirmed its enhanced apoptotic effect [131]. Overall, the results demonstrate that CM-NPs exhibit stronger anti-tumor activity than free curcumin in U87 MG cells, making it a promising candidate for glioblastoma therapy.

Jiang et al. explored the use of curcumin-loaded zeolite Y nanoparticles incorporated into polycaprolactone/gelatin electrospun nanofibers for the post-surgical treatment of glioblastoma [132]. The researchers successfully fabricated these nanofibers using electrospinning and confirmed the incorporation of zeolite Y nanoparticles [132]. The characterization of the nanofibers revealed suitable physical and chemical properties for biomedical applications [132]. A key finding was the sustained release of curcumin from the nanofibers, which is crucial for maintaining therapeutic drug levels at the tumor site [132]. In vitro studies demonstrated the efficacy of these nanofibers in inhibiting glioblastoma cell proliferation, suggesting their potential in reducing tumor recurrence [132]. Importantly, the nanofibers were found to be biocompatible, paving the way for future in vivo studies [132].

Zhang et al. developed curcumin-loaded zein nanoparticles functionalized with a G23 peptide (CUR-ZpD-G23 NPs) for enhanced glioblastoma treatment [133]. These ~120 nm spherical nanoparticles demonstrated improved blood–brain barrier penetration and tumor spheroid infiltration [133]. The G23 peptide facilitated enhanced cellular uptake by C6 glioma cells and increased transcytosis across an in vitro BBB model [133]. The nanoparticles exhibited concentration-dependent cytotoxicity in C6 glioma cells, inhibiting cell migration and colony formation while increasing reactive oxygen species production and inducing apoptosis [133]. In vivo studies using zebrafish models demonstrated stable circulation of the nanoparticles without aggregation [133]. While these findings are promising, further in vivo glioblastoma studies are needed to validate the efficacy and safety of CUR-ZpD-G23 NPs for glioblastoma therapy.

Şentürk et al. studied the development and evaluation of GRGDS-conjugated (glycine-arginine-glycine-aspartic acid-serine) and curcumin-loaded magnetic polymeric nanoparticles for targeted hyperthermia treatment of glioblastoma [134]. The nanoparticles were designed to deliver curcumin, conjugated with the GRGDS peptide for targeted binding to glioblastoma cells, and incorporated magnetic properties for external magnetic field guidance to the tumor site [134]. This study explored the use of these nanoparticles in hyperthermia treatment, utilizing an external magnetic field to induce heat and enhance curcumin’s therapeutic effect [134]. The results demonstrated the effective targeting of glioblastoma cells by the GRGDS-conjugated, curcumin-loaded nanoparticles [134]. The combined targeted drug delivery and hyperthermia approach significantly reduced cancer cell viability [134]. This dual-action strategy holds promise for improving treatment outcomes in glioblastoma by minimizing damage to healthy cells while enhancing therapeutic efficacy.

In their study, Javed et al. investigated the efficacy of curcumin-loaded lignin-g-p gold nanogels against glioblastoma [135]. The synthesized nanogels were characterized as spherical with a size of approximately 180 nm, ideal for cellular uptake and drug delivery [135]. These curcumin-loaded nanogels exhibited significant anti-cancer activity against U-251 MG GBM cells, with an IC50 value of 30 μM [135]. The mechanism of action involved apoptosis induction, confirmed by the expression of caspase-3 and cleaved caspase-3 proteins [135]. This kinetic drug release study demonstrated a controlled release profile, with up to 86% curcumin release (combined with piperine) within 250 min at a pH of 4 [135]. Importantly, the nanogels showed enhanced cellular internalization compared to gold nanoparticles or nanogels alone, suggesting improved therapeutic potential [135].

The study by Arzani et al. investigated curcumin-loaded poly(lactic-co-glycolic acid) nanoparticles (CUR-PLGA-NPs) as a means to improve curcumin’s bioavailability for cancer treatment [136]. The nanoparticles were prepared using a single emulsion method and characterized using dynamic light scattering, scanning electron microscopy, and differential scanning calorimetry (DSC) [136]. The results showed an encapsulation efficiency of 89.77% and a loading content of 9.06% [136]. Curcumin release from the nanoparticles exhibited a biphasic profile, with an initial burst release followed by sustained release [136]. DSC analysis confirmed the amorphous dispersion of curcumin within the nanoparticles [136]. Cytotoxicity studies on U87MG glioblastoma cells revealed that CUR-PLGA-NPs were more cytotoxic than free curcumin, with IC50 values of 32.90 μg/mL and 57.99 μg/mL, respectively, after 72 h [136]. These findings suggest that CUR-PLGA-NPs offer a promising drug delivery system for enhancing curcumin’s therapeutic efficacy in cancer treatment.

Research by Maiti et al. has shown that solid lipid curcumin particles (SLCPs) enhance curcumin’s bioavailability and anti-cancer effects against glioblastoma compared to natural curcumin due to improved solubility and stability [137]. In vitro studies using 20 μM SLCPs on U-87MG and U-251MG glioblastoma cell lines demonstrated significant cell death and inhibited proliferation, with enhanced effects observed when combined with berberine [137]. SLCP treatment induced apoptosis, evidenced by increased DNA fragmentation [137]. Additionally, SLCPs disrupted mitochondrial function, leading to decreased mitochondrial membrane potential and ATP levels [137]. Treatment also increased reactive oxygen species production, contributing to oxidative stress and cell death [137]. Importantly, SLCPs inhibited the PI3K/Akt/mTOR signaling pathway, a critical pathway for cell growth and survival, with enhanced inhibition observed in combination with berberine [137]. These findings suggest the potential of SLCPs, particularly in combination therapies, for glioblastoma treatment.

A study comparing the effects of curcumin and solid lipid curcumin particles on autophagy and mitophagy in glioblastoma multiforme cells showed that SLCPs demonstrated superior induction of autophagy markers (Atg5, Atg7, Beclin-1, and LC3A/B) compared to Cur in U-87MG, GL261, and F98 GBM cell lines [138]. This effect was selective to GBM cells, with minimal impact observed in rat C6-glioma and mouse N2a cells [138]. Furthermore, SLCPs exhibited a stronger inhibition of mitophagy markers (BNIP3L/NIX, FUNDC1, BNIP3, PINK-1, and HIF-1α) than Cur in GBM cells [138]. Both Cur and SLCPs inhibited the PI3K-Akt/mTOR pathway, crucial for cell growth and proliferation, with SLCPs demonstrating greater potency [138]. Chaperone-mediated autophagy was also affected, with both treatments downregulating LAMP2A and increasing HSP70, while HSC70 remained unchanged [138]. SLCP treatment resulted in a higher number of autophagy vacuoles in U-87MG cells compared to Cur [138]. These findings suggest that SLCPs may be a more effective therapeutic agent than natural Cur for GBM by modulating autophagy, mitophagy, and the PI3K-Akt/mTOR pathway.

Yeo et al. also aimed to improve curcumin’s bioavailability using a solid lipid nanoparticle system [139]. Curcumin-loaded SLNs were prepared via sonication and characterized using UV-Vis (ultraviolet–visible)and FTIR (Fourier Transform Infrared Spectroscopy) spectroscopy [139]. Particle size varied depending on the lipid used: stearic acid (14.70–149.30 nm), lauric acid (502.83 nm), and palmitic acid (469.53 nm) [139]. These size differences were attributed to varying chemical interactions (hydrogen bonding and van der Waals forces) between curcumin and the lipids, with stronger van der Waals forces resulting in smaller, more compact particles [139]. The Cur-loaded SLNs exhibited enhanced cytotoxicity against HeLa, A549, and CT-26 cancer cell lines compared to free curcumin, suggesting improved anti-cancer efficacy [139]. The anti-cancer effect was dependent on both particle size and cell line [139]. These findings highlight the potential of Cur-loaded SLNs for anti-cancer therapy due to their enhanced bioavailability and cytotoxic effects.

Wang et al. explored the co-delivery of curcumin and camptothecin (CPT) using neurotransmitter analog-modified liposomes to enhance glioma treatment [140]. The combination of CUR and CPT was found to downregulate the CPT-induced overexpression of programmed cell death 1 ligand 1, preventing T-cell inactivation and improving chemo-immunotherapy effectiveness [140]. Both CUR and the lipidized tryptamine in the liposomes interfered with the indoleamine 2,3-dioxygenase pathway, reducing regulatory T cell-mediated immunosuppression and enhancing anti-tumor immunity [140]. Combining CUR with PD-L1 inhibition offered synergistic potential in boosting anti-tumor immunity and alleviating the immunosuppressive glioma microenvironment [140]. The multifunctional nanoparticle platform not only facilitated targeted drug delivery across the BBB but also contributed to mitigating immunosuppression, a significant hurdle in glioma therapy [140].

## 6. Conclusions

The prognosis for glioblastoma remains dismal, with standard temozolomide and radiation therapy unchanged for over a decade, highlighting the urgent need for advancements in treatment. Curcumin’s modulation of multiple pathways, including oxidative stress, Pl3K/Akt, NF-κB, JAK/STAT, p53, MAPK, and Shh, underscores its multifaceted anti-cancer potential and presents an attractive potential adjunct to current therapies. Despite limited in vivo data, in vitro evidence suggests curcumin’s potential, by itself and in combination therapy, as a safe and effective anti-glioblastoma agent. However, curcumin’s poor bioavailability and pharmacokinetics pose a significant barrier to systemic use. Studies have shown potential in the use of its analogs and developing novel delivery systems to enhance its clinical applicability. While the results are promising, further large-scale studies and clinical trials are crucial to determine its role in glioblastoma treatment.

## Figures and Tables

**Table 2:** Novel curcumin delivery systems.

Study	Cell Line	Novel Delivery System	Key Findings	References
Keshavarz et al.	U87-MG	Dendrosomal Nanocurcumin + p53 overexpression	-DNC inhibits cell proliferation in a time- and dose-dependent manner. -Combined treatment significantly increases apoptosis (90%) compared to DNC alone (38%) or p53 overexpression alone (15%). -Combined treatment enhances GADD45 expression and reduces NF-κB and c-Myc expression.	[122]
Tondro et al.	U87	Nanocurcumin vs. free curcumin	-Both nanocurcumin and free curcumin reduce IL6 and TNF-α secretion. -Nanocurcumin exhibits superior efficacy in inhibiting cytokine production compared to free curcumin.	[123]
Hesari et al.	GBM cells	Nanomicelle curcumin	-Decreases p65 expression, a key subunit of the NF-κB complex. -Leads to decreased tumor cell proliferation and increased apoptosis.	[95]
Bagherian et al.	U87	Curcumin, nanomicellar-curcumin, temozolomide, and combinations	-All treatments (except 20 μM curcumin alone) significantly reduced cell viability. -Curcumin (50 μM), nanomicellar-curcumin, and the combination of nanomicellar-curcumin and TMZ significantly inhibited cell invasion and migration. -Increased levels of autophagy-related proteins (Beclin 1, LC3-I, and LC3-II). -Promoted apoptosis (increased Bcl-2 and caspase 8, decreased Bax). -Downregulated genes associated with the Wnt signaling pathway (β-catenin, cyclin D1, Twist, and ZEB1).	[124]
He et al.	GL261	Curcumin/Fa-PEG-PLA nanoparticles	-Superior efficacy in suppressing cell growth compared to free curcumin and Cur/MPEG-PLA. -Enhanced apoptosis induction. -In vivo: repressed tumor growth in subcutaneous and intracranial models by suppressing angiogenesis and promoting apoptosis.	[125]
Liang et al.	N/A	Cur/TMZ nanogel	-Excellent drug-loading capacity and sustained drug release. -Effectively inhibited the recurrence of TMZ-resistant tumors. -Low drug-induced toxicity. -Maintained Cur/TMZ ratio for consistent synergistic effects.	[126]
Ghoreyshi et al.	N/A	Curcumin nanoparticles	-Reduced intracellular reactive oxygen species and malondialdehyde levels (reduced oxidative stress). -Increased gene expression and activity of antioxidant enzymes (superoxide dismutase, catalase, glutaredoxin, thioredoxin).	[67]
Gallien et al.	Mouse, rat (F98), human (U87)	Curcumin-loaded dendrimer (G4 90/10-Cys-Cur)	-Significantly reduced viability of all three glioblastoma cell lines compared to non-cancerous control cells. -Unencapsulated curcumin did not show similar efficacy.	[127]
Hou et al.	N/A	Curcumin-loaded poloxamer188-based nanoparticles (P188TT NPs)	-Faster curcumin release at pH 6.8 (tumor microenvironment) than at pH 7.4. -Good brain-targeting efficiency. -Enhanced curcumin uptake in glioma cells and increased anti-tumor activity.	[128]
Schulze et al.	U87	Curcumin-loaded lipid nanoparticles + photodynamic therapy	-Enhanced photodynamic therapy against glioblastoma in a chorioallantois membrane model.	[129]
Negah et al.	N/A	Curcumin-loaded niosome nanoparticles	-Enhanced anti-tumor effects against glioblastoma stem-like cells compared to free curcumin. -Significant reduction in cell viability, proliferation, and migration of GSCs. -Higher levels of apoptosis and cell cycle arrest in GSCs. -Increased Bax expression and decreased Bcl2 expression. -Significant increase in reactive oxygen species production in GSCs. -Impaired GSC migration and invasiveness, potentially through MCP-1-mediated pathways. -Reduced secretion of MMP-2.	[130]
Tondro et al.	U87 MG	Curcumin-loaded niosome nanoparticles vs. free curcumin	-Both CM and CM-NPs reduced cell proliferation, but CM-NPs induced significantly higher levels of apoptosis. -CM-NPs exhibited superior inhibition of cell migration. -Significant increase in reactive oxygen species production with CM-NPs. -CM-NPs downregulated NF-κB and STAT3 expression and reduced IL-1β and TGF-β production. -Increased DNA fragmentation in U87 cells treated with CM-NPs.	[131]
Jiang et al.	N/A	Curcumin-loaded zeolite Y nanoparticles incorporated into polycaprolactone/gelatin electrospun nanofibers	-Sustained release of curcumin from the nanofibers. -Inhibited glioblastoma cell proliferation in vitro. -Nanofibers were biocompatible.	[132]
Zhang et al.	C6	Curcumin-loaded zein nanoparticles functionalized with a G23 peptide (CUR-ZpD-G23 NPs)	-Improved blood–brain barrier penetration and tumor spheroid infiltration. -Enhanced cellular uptake by C6 glioma cells and increased transcytosis across an in vitro BBB model. -Concentration-dependent cytotoxicity in C6 glioma cells, inhibiting cell migration and colony formation. -Increased reactive oxygen species production and induced apoptosis. -Stable circulation of the nanoparticles without aggregation in zebrafish models.	[133]
Şentürk et al.	N/A	GRGDS-conjugated and curcumin-loaded magnetic polymeric nanoparticles	-Effective targeting of glioblastoma cells. -Combined targeted drug delivery and hyperthermia treatment significantly reduced cancer cell viability.	[134]
Javed et al.	U-251 MG	Curcumin-loaded lignin-g-p gold nanogels	-Significant anti-cancer activity with an IC50 value of 30 μM. -Apoptosis induction (caspase-3 and cleaved caspase-3 expression). -Controlled release profile (up to 86% curcumin release within 250 min at pH 4). -Enhanced cellular internalization compared to gold nanoparticles or nanogels alone.	[135]
Arzani et al.	U87MG	Curcumin-loaded poly(lactic-co-glycolic acid) nanoparticles	-Encapsulation efficiency of 89.77% and loading content of 9.06%. -Biphasic release profile (initial burst followed by sustained release). -Amorphous dispersion of curcumin within the nanoparticles. -Higher cytotoxicity than free curcumin (IC50 values of 32.90 μg/mL vs. 57.99 μg/mL after 72 h).	[136]
Maiti et al.	U-87MG, U-251MG	Solid lipid curcumin particles	-Enhanced bioavailability and anti-cancer effects compared to natural curcumin. -Significant cell death and inhibited proliferation. -Enhanced effects when combined with berberine. -Induced apoptosis (increased DNA fragmentation). -Disrupted mitochondrial function (decreased mitochondrial membrane potential and ATP levels). -Increased reactive oxygen species production. -Inhibited the PI3K/Akt/mTOR signaling pathway.	[137]
Maiti et al.	U-87MG, GL261, F98	Solid lipid curcumin particles vs. curcumin	-SLCPs demonstrated superior induction of autophagy markers compared to Cur. -Stronger inhibition of mitophagy markers by SLCPs than Cur in GBM cells. -Both inhibited the PI3K-Akt/mTOR pathway, with SLCPs showing greater potency. -SLCP treatment resulted in a higher number of autophagy vacuoles in U-87MG cells.	[138]
Yeo et al.	HeLa, A549, CT-26	Curcumin-loaded solid lipid nanoparticles	-Enhanced cytotoxicity compared to free curcumin. -Particle size varied depending on the lipid used. -Anti-cancer effect dependent on particle size and cell line.	[139]
Wang et al.	N/A	Co-delivery of curcumin and camptothecin using neurotransmitter analog-modified liposomes	-Downregulated CPT-induced PD-L1 overexpression, preventing T-cell inactivation and improving chemo-immunotherapy efficacy. -Interfered with the indoleamine 2,3-dioxygenase pathway, reducing regulatory T cell-mediated immunosuppression. -Facilitated targeted drug delivery across the blood–brain barrier and mitigated immunosuppression in the glioma microenvironment.	[140]

## Data Availability

No new data were created or analyzed in this study. Data sharing is not applicable to this article.

## References

[1] Torp S.H., Solheim O., Skjulsvik A.J. (2022). The WHO 2021 Classification of Central Nervous System Tumours: A Practical Update on What Neurosurgeons Need to Know—A Minireview. Acta Neurochir..

[2] Price M., Pittman Ballard C.A., Benedetti J., Neff C., Cioffi G., Waite K., Kruchko C., Barnholtz-Sloan J.S., Ostrom Q.T. (2024). CBTRUS Statistical Report: Primary Brain and Other Central Nervous System Tumors Diagnosed in the United States in 2017–2021. Neuro-Oncol..

[3] Lan Z., Li X., Zhang X. (2024). Glioblastoma: An Update in Pathology, Molecular Mechanisms and Biomarkers. Int. J. Mol. Sci..

[4] Miller K.D., Ostrom Q.T., Kruchko C., Patil N., Tihan T., Cioffi G., Fuchs H.E., Waite K., Jemal A., Siegel R.L. (2021). Brain and Other Central Nervous System Tumor Statistics, 2021. CA A Cancer J. Clin..

[5] Sejda A., Grajkowska W., Trubicka J., Szutowicz E., Wojdacz T.K., Kloc W., Izycka-Swieszewska E. (2022). WHO CNS5 2021 Classification of Gliomas: A Practical Review and Road Signs for Diagnosing Pathologists and Proper Patho-Clinical and Neuro-Oncological Cooperation. Folia Neuropathol..

[6] Bijalwan G., Shrivastav A.K., Mallik S., Dubey M.K. (2024). Glioblastoma Multiforme—A Rare Type of Cancer: A Narrative Review. Cancer Res. Stat. Treat..

[7] Bruhn H., Tavelin B., Rosenlund L., Henriksson R. (2024). Do Presenting Symptoms Predict Treatment Decisions and Survival in Glioblastoma? -Real World Data from 1458 Patients in the Swedish Brain Tumour Registry. Neuro-Oncol. Pract..

[8] Sekely A., Bernstein L.J., Campbell K.L., Mason W.P., Laperriere N., Kalidindi N., Or R., Ramos R., Climans S.A., Pond G.R. (2022). Neurocognitive Impairment, Neurobehavioral Symptoms, Fatigue, Sleep Disturbance, and Depressive Symptoms in Patients with Newly Diagnosed Glioblastoma. Neuro-Oncol. Pract..

[9] Bruhn H., Rosenlund L., Tavelin B., Henriksson R. (2023). Os13.7.a Onset Symptoms Predict Survival in Glioblastoma Patients-Real World Data from 1719 Patients in the Swedish Brain Tumour Registry 2018–2021. Neuro-Oncol..

[10] Mrowczynski O.D., Yang A.L., Liao J., Rizk E. (2021). The Potential of Glioblastoma Patient Symptoms to Diagnose and Predict Survival. Cureus.

[11] Bian Y.L., Wang Y., Chen X., Zhang Y., Xiong S., Su D. (2023). Image-guided Diagnosis and Treatment of Glioblastoma. VIEW.

[12] Alipourfard I., Alivirdiloo V., Hashemi S.B., Yazdani Y., Ghazi F., Eslami M., Ameri Shah Reza M., Dadashpour M. (2023). Recent Advances in the Detection of Glioblastoma, from Imaging-Based Methods to Proteomics and Biosensors: A Narrative Review. Cancer Cell Int..

[13] Aldecoa I., Archilla I., Ribalta T. (2023). Practice guidelines for the diagnosis of glioblastoma. New Insights Into Glioblastoma.

[14] Seyhan A.A. (2024). Circulating Liquid Biopsy Biomarkers in Glioblastoma: Advances and Challenges. Int. J. Mol. Sci..

[15] Linhares P., Carvalho B., Vaz R., Costa B.M. (2020). Glioblastoma: Is There Any Blood Biomarker with True Clinical Relevance?. Int. J. Mol. Sci..

[16] Nag A., Sachithanandam S.V., Lucke-Wold B. (2024). Predictive and Prognostic Significance of Molecular Biomarkers in Glioblastoma. Biomedicines.

[17] Yıldırım Ö., Önay Uçar E. (2024). The Molecular Landscape of Glioblastoma: Implications for Diagnosis and Therapy. Eur. J. Biol..

[18] Świątek W., Kłodziński O., Ciesielski M., Adamkiewicz Z., Podolak M., Mozdziak P., Kranc W. (2024). Glioblastoma: A Molecular Insight into Current Discoveries and Treatment Directions. Med. J. Cell Biol..

[19] Rabah N., Ait Mohand F.-E., Kravchenko-Balasha N. (2023). Understanding Glioblastoma Signaling, Heterogeneity, Invasiveness, and Drug Delivery Barriers. Int. J. Mol. Sci..

[20] Obrador E., Moreno-Murciano P., Oriol-Caballo M., López-Blanch R., Pineda B., Gutiérrez-Arroyo J.L., Loras A., Gonzalez-Bonet L.G., Martinez-Cadenas C., Estrela J.M. (2024). Glioblastoma Therapy: Past, Present and Future. Int. J. Mol. Sci..

[21] Thakur A., Faujdar C., Sharma R., Sharma S., Malik B., Nepali K., Liou J.P. (2022). Glioblastoma: Current Status, Emerging Targets, and Recent Advances. J. Med. Chem..

[22] Polonara G., Aiudi D., Iacoangeli A., Raggi A., Ottaviani M.M., Antonini R., Iacoangeli M., Dobran M. (2023). Glioblastoma: A Retrospective Analysis of the Role of the Maximal Surgical Resection on Overall Survival and Progression Free Survival. Adv. Cardiovasc. Dis..

[23] Ishaque A., Das S. (2024). Cutting Through History: The Evolution of Glioblastoma Surgery. Curr. Oncol..

[24] Shah S. (2024). Novel Therapies in Glioblastoma Treatment: Review of Glioblastoma; Current Treatment Options; and Novel Oncolytic Viral Therapies. Med. Sci..

[25] Aziz P.A., Memon S., Al Mubarak H.M., Memon A.S., Abbas K., Qazi S.U., Memon R.A., Qambrani K.A., Taj O., Ghazanfar S. (2024). Surg-10. Supratotal Resection: An Emerging Concept of Glioblastoma Multiforme Surgery—Systematic Review and Meta-Analysis. Neuro-Oncol..

[26] Patel V., Chavda V. (2023). Intraoperative Glioblastoma Surgery-Current Challenges and Clinical Trials: An Update. Cancer Pathog. Ther..

[27] Osawa S., Fujita S., Tsuchiya T., Yanagisawa S., Ohno M., Takahashi M., Narita Y. (2023). 10115-Stmo-4 Surgical Outcomes of Awake Surgery for Glioblastoma. Neuro-Oncol. Adv..

[28] Li N., Hao S., Cao X., Lin Y., Li Y., Dai T., Liu M. (2024). Significance of Radiation Therapy in Frontal Glioblastoma Patients and Exploration of Optimal Treatment Modality: A Real-World Multiple-Center Study Based on Propensity Score Matching. Quant. Imaging Med. Surg..

[29] Jing B., Sun R., Pan Z., Wei S. (2024). Current Chemotherapy Strategies for Adults with IDH-Wildtype Glioblastoma. Front. Oncol..

[30] Hoosemans L., Vooijs M.A., Hoeben A. (2024). Opportunities and Challenges of Small Molecule Inhibitors in Glioblastoma Treatment: Lessons Learned from Clinical Trials. Cancers.

[31] Duan M., Cao R., Yang Y., Chen X., Liu L., Ren B., Wang L., Goh B.-C. (2024). Blood–Brain Barrier Conquest in Glioblastoma Nanomedicine: Strategies, Clinical Advances, and Emerging Challenges. Cancers.

[32] Shah S., Mansour H.M., Aguilar T.M., Lucke-Wold B. (2024). Advances in Anti-Cancer Drug Development: Metformin as Anti-Angiogenic Supplemental Treatment for Glioblastoma. Int. J. Mol. Sci..

[33] Farooq M., Scalia G., Umana G.E., Parekh U.A., Naeem F., Abid S.F., Khan M.H., Zahra S.G., Sarkar H.P., Chaurasia B. (2023). A Systematic Review of Nanomedicine in Glioblastoma Treatment: Clinical Efficacy, Safety, and Future Directions. Brain Sci..

[34] Persano F., Gigli G., Leporatti S. (2022). Natural Compounds as Promising Adjuvant Agents in The Treatment of Gliomas. Int. J. Mol. Sci..

[35] Valerius A.R., Webb L.M., Thomsen A., Lehrer E.J., Breen W.G., Campian J.L., Riviere-Cazaux C., Burns T.C., Sener U. (2024). Review of Novel Surgical, Radiation, and Systemic Therapies and Clinical Trials in Glioblastoma. Int. J. Mol. Sci..

[36] Belue M.J., Harmon S.A., Chappidi S., Zhuge Y., Taşçı E., Jagasia S., Joyce T., Camphausen K., Türkbey B., Krauze A. (2024). Diagnosing Progression in Glioblastoma—Tackling a Neuro-Oncology Problem Using Artificial-Intelligence-Derived Volumetric Change over Time on Magnetic Resonance Imaging to Examine Progression-Free Survival in Glioblastoma. Diagnostics.

[37] Dhanavath N., Bisht P., Jamadade M.S., Murti K., Wal P., Kumar N. (2024). Olaparib: A Chemosensitizer for the Treatment of Glioblastoma. Mini Rev. Med. Chem..

[38] Chen Q., Wu D., Chen Z. (2023). Mechanical Nanosurgery Approach: Assistance to Overcome the Chemotherapy Resistance of Glioblastoma. MedComm.

[39] Sadowski K., Jażdżewska A., Kozłowski J., Zacny A., Lorenc T., Olejarz W. (2024). Revolutionizing Glioblastoma Treatment: A Comprehensive Overview of Modern Therapeutic Approaches. Int. J. Mol. Sci..

[40] Kaur K., Al-Khazaleh A.K., Bhuyan D.J., Li F., Li C.G. (2024). A Review of Recent Curcumin Analogues and Their Antioxidant, Anti-Inflammatory, and Anticancer Activities. Antioxidants.

[41] Gupta B., Sharma P.K., Malviya R., Mishra P.S. (2024). Curcumin and Curcumin Derivatives for Therapeutic Applications: In Vitro and In Vivo Studies. Curr. Nutr. Food Sci..

[42] Pandey P.O., Ali B., Mishra A. (2022). Curcumin: A Pharmacologically Functional Active Ingredient from Nature. Int. J. Adv. Acad. Stud..

[43] Buniowska-Olejnik M., Mykhalevych A., Urbański J., Berthold-Pluta A., Michałowska D., Banach M. (2024). The Potential of Using Curcumin in Dairy and Milk-Based Products—A Review. J. Food Sci..

[44] Singh A., Soni U., Varadwaj P.K., Misra K., Rizvi S.I. (2023). Anti-Inflammatory Effect of Curcumin in an Accelerated Senescence Model of Wistar Rat: An in Vivo and in-Silico Study. J. Biomol. Struct. Dyn..

[45] Rapti E., Adamantidi T., Efthymiopoulos P., Kyzas G.Z., Τσούπρας A. (2024). Potential Applications of the Anti-Inflammatory, Antithrombotic and Antioxidant Health-Promoting Properties of Curcumin: A Critical Review. Nutraceuticals.

[46] Hu Y., Cheng L., Du S., Wang K., Liu S. (2023). Antioxidant Curcumin Induces Oxidative Stress to Kill Tumor Cells (Review). Oncol. Lett..

[47] Roman B., Retajczyk M., Sałaciński Ł., Pełech R. (2020). Curcumin-Properties, Applications and Modification of Structure. Mini-Rev. Org. Chem..

[48] Urosevic M., Nikolić L., Savic Gajic I.M., Nikolić V., Dinić A., Miljković V. (2022). Curcumin: Biological Activities and Modern Pharmaceutical Forms. Antibiotics.

[49] Osifová Z., Reiberger R., Císařová I., Machara A., Dračínský M. (2022). Diketo-Ketoenol Tautomers in Curcuminoids: Synthesis, Separation of Tautomers, and Kinetic and Structural Studies. J. Org. Chem..

[50] Chatterjee P., Dutta S., Chakraborty T. (2022). Tautomers and Rotamers of Curcumin: A Combined UV Spectroscopy, High-Performance Liquid Chromatography, Ion Mobility Mass Spectrometry, and Electronic Structure Theory Study. J. Phys. Chem. A.

[51] Heger M., van Golen R.F., Broekgaarden M., Michel M.C. (2014). The Molecular Basis for the Pharmacokinetics and Pharmacodynamics of Curcumin and Its Metabolites in Relation to Cancer. Pharmacol. Rev..

[52] Hatamipour M., Johnston T.P., Sahebkar A. (2018). One Molecule, Many Targets and Numerous Effects: The Pleiotropy of Curcumin Lies in Its Chemical Structure. Curr. Pharm. Des..

[53] da Silva Lopes L., Pereira S.K.S., Lima L.K.F., Rai M., Feitosa C.M. (2023). Pharmacokinetics and Pharmacodynamics of Curcumin. Curcumin and Neurodegenerative Diseases.

[54] Niwa T., Yokoyama S., Mochizuki M., Osawa T. (2019). Curcumin Metabolism by Human Intestinal Bacteria in Vitro. J. Funct. Foods.

[55] Shi M., Gao T., Zhang T., Han H. (2019). Characterization of Curcumin Metabolites in Rats by Ultra-High-Performance Liquid Chromatography with Electrospray Ionization Quadrupole Time-of-Flight Tandem Mass Spectrometry. Rapid Commun. Mass Spectrom..

[56] Hassaninasab A., Hashimoto Y., Tomita-Yokotani K., Kobayashi M. (2011). Discovery of the Curcumin Metabolic Pathway Involving a Unique Enzyme in an Intestinal Microorganism. Proc. Natl. Acad. Sci. USA.

[57] Bresciani L., Favari C., Calani L., Francinelli V., Riva A., Petrangolini G., Allegrini P., Mena P., Del Rio D. (2020). The Effect of Formulation of Curcuminoids on Their Metabolism by Human Colonic Microbiota. Molecules.

[58] Jithavech P., Ratnatilaka Na Bhuket P., Supasena W., Qiu G., Ye S., Wu J., Wong T.W., Rojsitthisak P. (2020). In Vitro Hepatic Metabolism of Curcumin Diethyl Disuccinate by Liver S9 from Different Animal Species. Front. Pharmacol..

[59] Nguyen H.D., Kim M.S. (2022). The Protective Effects of Curcumin on Metabolic Syndrome and Its Components: In-Silico Analysis for Genes, Transcription Factors, and microRNAs Involved. Arch. Biochem. Biophys..

[60] Adiwidjaja J., Boddy A.V., McLachlan A.J. (2020). Physiologically-Based Pharmacokinetic Predictions of the Effect of Curcumin on Metabolism of Imatinib and Bosutinib: In Vitro and In Vivo Disconnect. Pharm. Res..

[61] Zahra M., Hadi F., Maqbool T., Sultana H., Abid F., Aslam M.A., Ahmad M., Muhammad S., Hassan M.Q.U. (2024). Curcumin (Turmeric): A Carcinogenic, Miscarriage and Cirrhosis Causing Agent. J. Health Rehabil. Res..

[62] Ardana T., Yuandani Y., Satria D., Putra E.D.L., Muhammad M., Rosidah R. (2024). Acute Toxicity Evaluation of Curcuma Domestica Vahl. Rhizome Vco Curcuminoid Extract. Int. J. Appl. Pharm..

[63] Mulyani Y., Hasimun P., Nurjanah S. (2022). Subcrhonic Toxicity of Curcuma Longa (Tumeric) Rhizoma Extract on Rats. Maj. Obat Tradis..

[64] Hamdy S., Elshopakey G.E., Risha E., Rezk S., Ateya A., Abdelhamid F.M. (2023). Curcumin Mitigates Gentamicin Induced-Renal and Cardiac Toxicity via Modulation of Keap1/Nrf2, NF-κB/iNOS and Bcl-2/BAX Pathways. Food Chem. Toxicol..

[65] Radwan A.M., Karhib M.M., Tousson E. (2023). Curcumin Alleviates Thioacetamide-Induced Kidney Toxicity in Rats: Enhancing Antioxidant System, and Attenuating Oxidative Stress, DNA Damage, and Inflammation. Biomed. Pharmacol. J..

[66] Jafari-Nozad A.M., Jafari A.M., Aschner M., Farkhondeh T., Samarghandian S. (2022). Curcumin Combats Against Organophosphate Pesticides Toxicity: A Review of the Current Evidence and Molecular Pathways. Curr. Med. Chem..

[67] Ghoreyshi N., Ghahremanloo A., Javid H., Homayouni Tabrizi M., Hashemy S.I. (2023). Effect of Folic Acid-Linked Chitosan-Coated PLGA-Based Curcumin Nanoparticles on the Redox System of Glioblastoma Cancer Cells. Phytochem. Anal..

[68] Lambring C.B., Chen L., Nelson C., Stevens A., Bratcher W., Basha R. (2023). Oxidative Stress and Cancer: Harnessing the Therapeutic Potential of Curcumin and Analogues Against Cancer. Eur. J. Biol..

[69] Trotta T., Panaro M.A., Prifti E., Porro C. (2019). Modulation of Biological Activities in Glioblastoma Mediated by Curcumin. Nutr. Cancer.

[70] Wei Y., Li H., Li Y., Yu Z., Quan T.P., Leng Y., Chang E., Bai Y., Bian Y., Hou Y. (2024). Advances of Curcumin in Nervous System Diseases: The Effect of Regulating Oxidative Stress and Clinical Studies. Front. Pharmacol..

[71] Rinaldi M., Caffo M., Minutoli L., Marini H., Abbritti R.V., Squadrito F., Trichilo V., Valenti A., Barresi V., Altavilla D. (2016). ROS and Brain Gliomas: An Overview of Potential and Innovative Therapeutic Strategies. Int. J. Mol. Sci..

[72] Snezhkina A.V., Kudryavtseva A.V., Kardymon O.L., Savvateeva M.V., Melnikova N.V., Krasnov G.S., Dmitriev A.A. (2019). ROS Generation and Antioxidant Defense Systems in Normal and Malignant Cells. Oxidative Med. Cell. Longev..

[73] Godoy P.R.D.V., Godoy P.R.D.V., Pour Khavari A., Rizzo M., Sakamoto-Hojo E.T., Haghdoost S., Haghdoost S. (2020). Targeting NRF2, Regulator of Antioxidant System, to Sensitize Glioblastoma Neurosphere Cells to Radiation-Induced Oxidative Stress. Oxidative Med. Cell. Longev..

[74] Orlicka-Płocka M., Fedoruk-Wyszomirska A., Gurda-Woźna D., Pawelczak P., Krawczyk P.A., Giel-Pietraszuk M., Framski G., Ostrowski T., Wyszko E. (2021). Implications of Oxidative Stress in Glioblastoma Multiforme Following Treatment with Purine Derivatives. Antioxidants.

[75] Liu J., Wang Z. (2015). Increased Oxidative Stress as a Selective Anticancer Therapy. Oxidative Med. Cell. Longev..

[76] Krawczynski K., Krawczynski K., Godlewski J., Godlewski J., Bronisz A., Bronisz A. (2020). Oxidative Stress-Part of the Solution or Part of the Problem in the Hypoxic Environment of a Brain Tumor. Antioxidants.

[77] Kim J.Y., Jung C.-W., Lee W., Kim H.J., Jeong H.J., Park M.-J., Jang W.I., Kim E.H. (2022). Interaction of Curcumin with Glioblastoma Cells via High and Low Linear Energy Transfer Radiation Therapy Inducing Radiosensitization Effects. J. Radiat. Res..

[78] Seyithanoglu M.H., Abdallah A., Kitiş S., Guler E.M., Kocyigit A., Dundar T.T., Papaker M.G. (2019). Investigation of Cytotoxic, Genotoxic, and Apoptotic Effects of Curcumin on Glioma Cells. Cell. Mol. Biol..

[79] Alkahtani S., AL-Johani N.S., Alarifi S., Afzal M. (2023). Cytotoxicity Mechanisms of Blue-Light-Activated Curcumin in T98G Cell Line: Inducing Apoptosis through ROS-Dependent Downregulation of MMP Pathways. Int. J. Mol. Sci..

[80] Öz A., Çelik Ö., Övey İ.S. (2017). Effects of Different Doses of Curcumin on Apoptosis, Mitochondrial Oxidative Stress and Calcium Ion Influx in DBRG Glioblastoma Cells. J. Cell. Neurosci. Oxidative Stress.

[81] Agca C.A. (2019). Homocysteine-Induced Damage of Cultured Glioblastoma Cells: Amelioration by Curcumin. Neurophysiology.

[82] Gersey Z.C., Rodriguez G.A., Barbarite E., Sanchez A., Walters W.M., Ohaeto K.C., Komotar R.J., Graham R.M. (2017). Curcumin Decreases Malignant Characteristics of Glioblastoma Stem Cells via Induction of Reactive Oxygen Species. BMC Cancer.

[83] Cholia R.P., Kumari S., Kumar S., Kaur M., Kaur M., Kumar R., Dhiman M., Mantha A.K. (2017). An in Vitro Study Ascertaining the Role of H_2_O_2_ and Glucose Oxidase in Modulation of Antioxidant Potential and Cancer Cell Survival Mechanisms in Glioblastoma U-87 MG Cells. Metab. Brain Dis..

[84] Yin H., Zhou Y., Wen C., Zhou C., Zhang W., Hu X., Wang L., You C., Shao J. (2014). Curcumin Sensitizes Glioblastoma to Temozolomide by Simultaneously Generating ROS and Disrupting AKT/mTOR Signaling. Oncol. Rep..

[85] Luo S.-M., Wu Y.-P., Huang L.-C., Huang S.-M., Hueng D.-Y. (2021). The Anti-Cancer Effect of Four Curcumin Analogues on Human Glioma Cells. OncoTargets Ther..

[86] Wong S.C., Kamarudin M.N.A., Naidu R. (2021). Anticancer Mechanism of Curcumin on Human Glioblastoma. Nutrients.

[87] Zoi V., Kyritsis A.P., Galani V., Lazari D., Sioka C., Voulgaris S., Alexiou G.A. (2024). The Role of Curcumin in Cancer: A Focus on the PI3K/Akt Pathway. Cancers.

[88] Aoki H., Takada Y., Kondo S., Kondo S., Kondo S., Sawaya R., Aggarwal B.B., Kondo Y. (2007). Evidence That Curcumin Suppresses the Growth of Malignant Gliomas in Vitro and in Vivo through Induction of Autophagy: Role of Akt and Extracellular Signal-Regulated Kinase Signaling Pathways. Mol. Pharmacol..

[89] Bonafé G.A., Boschiero M.N., Sodré A.R., Ziegler J., Rocha T., Ortega M.M. (2022). Natural Plant Compounds: Does Caffeine, Dipotassium Glycyrrhizinate, Curcumin, and Euphol Play Roles as Antitumoral Compounds in Glioblastoma Cell Lines?. Front. Neurol..

[90] Mejía-Rodríguez R., Romero-Trejo D., González R.O., Segovia J.C. (2023). Combined Treatments with AZD5363, AZD8542, Curcumin or Resveratrol Induce Death of Human Glioblastoma Cells by Suppressing the PI3K/AKT and SHH Signaling Pathways. Biochem. Biophys. Rep..

[91] Su R.-Y., Hsueh S.-C., Chen C.Y., Hsu M.-J., Lu H.-F., Peng S.-F., Chen P.-Y., Lien J.-C., Chen Y.-L., Chueh F.-S. (2021). Demethoxycurcumin Suppresses Proliferation, Migration, and Invasion of Human Brain Glioblastoma Multiforme GBM 8401 Cells via PI3K/Akt Pathway. Anticancer Res..

[92] Chen C.-J., Shang H.S., Huang Y.-L., Tien N., Chen Y.-L., Hsu S.-Y., Wu R.S.-C., Tang C.-L., Lien J.-C., Lee M.-H. (2022). Bisdemethoxycurcumin Suppresses Human Brain Glioblastoma Multiforme GBM 8401 Cell Migration and Invasion via Affecting NF-κB and MMP-2 and MMP-9 Signaling Pathway in Vitro. Environ. Toxicol..

[93] Afshari A.R., Jalili-Nik M., Abbasinezhad-Moud F., Javid H., Karimi M.H., Mollazadeh H., Jamialahmadi T., Sathyapalan T., Sahebkar A. (2020). Anti-Tumor Effects of Curcuminoids in Glioblastoma Multiforme: An Updated Literature Review. Curr. Med. Chem..

[94] Uddin S., Kabir T., Al Mamun A., Sarwar S., Nasrin F., Nasrin F., Bin Emran T., Alanazi I.S., Rauf A., Albadrani G.M. (2021). Natural Small Molecules Targeting NF-κB Signaling in Glioblastoma. Front. Pharmacol..

[95] Hesari A., Rezaei M., Rezaei M., Dashtiahangar M., Fathi M., Ganji Rad J., Momeni F., Avan A., Ghasemi F. (2019). Effect of Curcumin on Glioblastoma Cells. J. Cell. Physiol..

[96] Ou A., Ott M., Fang D., Heimberger A.B. (2021). The Role and Therapeutic Targeting of JAK/STAT Signaling in Glioblastoma. Cancers.

[97] Ashrafizadeh M., Rafiei H., Mohammadinejad R., Ghasemipour Afshar E., Farkhondeh T., Samarghandian S. (2020). Potential Therapeutic Effects of Curcumin Mediated by JAK/STAT Signaling Pathway: A Review. Phytother. Res..

[98] Weissenberger J., Priester M., Bernreuther C., Rakel S., Glatzel M., Seifert V., Kögel D. (2010). Dietary Curcumin Attenuates Glioma Growth in a Syngeneic Mouse Model by Inhibition of the JAK1,2/STAT3 Signaling Pathway. Clin. Cancer Res..

[99] Fahmideh H., Shapourian H., Moltafeti R., Tavakol C., Forghaniesfidvajani R., Zalpoor H., Nabi-Afjadi M. (2022). The Role of Natural Products as Inhibitors of JAK/STAT Signaling Pathways in Glioblastoma Treatment. Oxidative Med. Cell. Longev..

[100] Hermawan A., Wulandari F., Hanif N., Utomo R.Y., Jenie R.I., Ikawati M., Tafrihani A.S. (2022). Identification of Potential Targets of the Curcumin Analog CCA-1.1 for Glioblastoma Treatment: Integrated Computational Analysis and in Vitro Study. Dent. Sci. Rep..

[101] Wang Z., Liu F., Liao W., Yu L., Hu Z., Li M., Xia H. (2020). Curcumin Suppresses Glioblastoma Cell Proliferation by P-AKT/mTOR Pathway and Increases the PTEN Expression. Arch. Biochem. Biophys..

[102] Oak S., Karajgikar O., Teni T. (2023). Curcumin Mediates Selective Aggregation of Mutant P53 in Cancer Cells: A Promising Therapeutic Strategy. Biochem. Biophys. Res. Commun..

[103] Garrido-Armas M., Corona J.C., Escobar M.L., Torres L., Ordóñez-Romero F., Hernández-Hernández A., Arenas-Huertero F. (2018). Paraptosis in Human Glioblastoma Cell Line Induced by Curcumin. Toxicol. Vitr..

[104] Wang P., Hao X., Li X., Yan Y., Tian W., Xiao L., Wang Z., Dong J. (2021). Curcumin Inhibits Adverse Psychological Stress-Induced Proliferation and Invasion of Glioma Cells via down-Regulating the ERK/MAPK Pathway. J. Cell. Mol. Med..

[105] Du W., Feng Y., Wang X., Piao X.-Y., Cui Y.-Q., Chen L., Lei X.-H., Sun X., Liu X., Wang H. (2013). Curcumin Suppresses Malignant Glioma Cells Growth and Induces Apoptosis by Inhibition of SHH/GLI1 Signaling Pathway in Vitro and Vivo. CNS Neurosci. Ther..

[106] Yin S., Du W., Wang F., Han B., Cui Y.-Q., Yang D., Chen H., Liu D., Liu X., Zhai X. (2018). MicroRNA-326 Sensitizes Human Glioblastoma Cells to Curcumin via the SHH/GLI1 Signaling Pathway. Cancer Biol. Ther..

[107] Zoi V., Galani V., Tsekeris P., Kyritsis A.P., Alexiou G.A. (2022). Radiosensitization and Radioprotection by Curcumin in Glioblastoma and Other Cancers. Biomedicines.

[108] Ryskalin L., Biagioni F., Busceti C.L., Lazzeri G., Frati A., Fornai F. (2020). The Multi-Faceted Effect of Curcumin in Glioblastoma from Rescuing Cell Clearance to Autophagy-Independent Effects. Molecules.

[109] Xiu Z., Sun T., Yang Y., He Y., Yang S., Xue X., Yang W.T. (2022). Curcumin Enhanced Ionizing Radiation-Induced Immunogenic Cell Death in Glioma Cells through Endoplasmic Reticulum Stress Signaling Pathways. Oxidative Med. Cell. Longev..

[110] Zoi V., Galani V., Vartholomatos E., Zacharopoulou N., Tsoumeleka E., Gkizas G., Bozios G., Tsekeris P., Chousidis I., Leonardos I. (2021). Curcumin and Radiotherapy Exert Synergistic Anti-Glioma Effect In Vitro. Biomedicines.

[111] Ghanbari B., Neshasteh Riz A., Hormozi Moghaddam Z.H.M. (2024). The Effect of Curcumin in Combination with Radiation Therapy and Hyperthermia for a Glioblastoma Spheroid Model. Internatuinal J. Radiat. Res..

[112] Wang W.H., Shen C.Y., Chien Y.C., Chang W.S., Tsai C.-W., Lin Y.H., Hwang J.J. (2020). Validation of Enhancing Effects of Curcumin on Radiotherapy with F98/FGT Glioblastoma-Bearing Rat Model. Int. J. Mol. Sci..

[113] Sminia P., van den Berg J., van Kootwijk A., Hageman E., Slotman B.J., Verbakel W.F.A.R. (2021). Experimental and clinical studies on radiation and curcumin in human glioma. J. Cancer Res. Clin. Oncol..

[114] Razali N.S.C., Lam K.W., Rajab N.F., Jamal A.R.A., Kamaluddin N.F., Chan K.M. (2022). Curcumin Piperidone Derivatives Induce Anti-Proliferative and Anti-Migratory Effects in LN-18 Human Glioblastoma Cells. Dent. Sci. Rep..

[115] Inai K., Hamabe-Horiike T., Ono M., Iwashimizu S., Ito R., Sunagawa Y., Katanasaka Y., Arakawa Y., Hasegawa K., Morimoto T. (2023). 10073-Cbms-6 Curcumin Analog b Exhibit Anti-Tumor Activity against Glioblastomaat Lower Concentrations than Curcumin. Neuro-Oncol. Adv..

[116] Inai K., Hamabe-Horiike T., Ono M., Iwashimizu S., Ito R., Sunagawa Y., Katanasaka Y., Hawke P., Arakawa Y., Morimoto T. (2023). Dddr-16. Curcumin Analogs Exhibit Anti-Tumor Activity against Glioblastoma at Lower Concentrations than Curcumin. Neuro-Oncol..

[117] Piwowarczyk L., Mlynarczyk D.T., Krajka-Kuźniak V., Majchrzak-Celińska A., Budzianowska A., Tomczak S., Budzianowski J., Woźniak-Braszak A., Pietrzyk R., Baranowski M. (2022). Natural Compounds in Liposomal Nanoformulations of Potential Clinical Application in Glioblastoma. Cancers.

[118] Bulnes S., Picó-Gallardo M., Bengoetxea H., Lafuente J.V. (2023). Effects of curcumin nanodelivery on schizophrenia and glioblastoma. Int. Rev. Neurobiol..

[119] Zhao C., Zhu X., Tan J., Mei C., Cai X., Kong F. (2024). Lipid-based nanoparticles to address the limitations of GBM therapy by overcoming the blood-brain barrier, targeting glioblastoma stem cells, and counteracting the immunosuppressive tumor microenvironment. Biomed. Pharmacother..

[120] Jnaidi R., Almeida A.J., Gonçalves L. (2020). Solid Lipid Nanoparticles and Nanostructured Lipid Carriers as Smart Drug Delivery Systems in the Treatment of Glioblastoma Multiforme. Pharmaceutics.

[121] Iturrioz-Rodríguez N., Bertorelli R., Ciofani G. (2021). Lipid-Based Nanocarriers for The Treatment of Glioblastoma. Adv. NanoBiomed Res..

[122] Keshavarz R., Bakhshinejad B., Babashah S., Baghi N., Sadeghizadeh M. (2016). Dendrosomal Nanocurcumin and P53 Overexpression Synergistically Trigger Apoptosis in Glioblastoma Cells. Iran. J. Basic Med. Sci..

[123] Tondro G., Rajabzade G., Mohammadi A., Moradi H., Negah S.S. (2022). Anti-Inflammatory Effects of Nano-Curcumin on a Glioblastoma Cell Line. Neurosci. J. Shefaye Khatam.

[124] Bagherian A., Mardani R., Roudi B., Taghizadeh M., Banfshe H.R., Ghaderi A., Davoodvandi A., Shamollaghamsari S., Hamblin M.R., Hamblin M.R. (2020). Combination Therapy with Nanomicellar-Curcumin and Temozolomide for In Vitro Therapy of Glioblastoma Multiforme via Wnt Signaling Pathways. J. Mol. Neurosci..

[125] He Y., Wu C., Duan J., Miao J., Ren H., Liu J. (2020). Anti-Glioma Effect with Targeting Therapy Using Folate Modified Nano-Micelles Delivery Curcumin. J. Biomed. Nanotechnol..

[126] Wu X., Zheng S., Zhuang J., Chen S. (2023). Curcumin Combining Temozolomide Formed Localized Nanogel for Inhibition of Postsurgical Chemoresistant Glioblastoma. Nanomedicine.

[127] Gallien J., Srinageshwar B., Gallo K., Holtgrefe G., Koneru S., Otero P.S., Bueno C.A., Mosher J., Roh A., Kohtz D.S. (2021). Curcumin Loaded Dendrimers Specifically Reduce Viability of Glioblastoma Cell Lines. Molecules.

[128] Hou X., Xu J., He S., Pan X., Yang J., Zhang N., Yang X. (2023). Curcumin-loaded Nanoparticle Based on Poloxamer188 for Glioma Treatment: Synthesis, Characterization and in Vitro Evaluation. Polym. Adv. Technol..

[129] Schulze J., Schöne L., Ayoub A.M., Librizzi D., Amin M.U., Engelhardt K., Yousefi B.H., Bender L., Schaefer J., Preis E. (2023). Modern Photodynamic Glioblastoma Therapy Using Curcumin- or Parietin-Loaded Lipid Nanoparticles in a CAM Model Study. ACS Appl. Bio Mater..

[130] Sahab-Negah S., Ariakia F., Jalili-Nik M., Afshari A.R., Salehi S., Samini F., Rajabzadeh G., Gorji A. (2020). Curcumin Loaded in Niosomal Nanoparticles Improved the Anti-Tumor Effects of Free Curcumin on Glioblastoma Stem-like Cells: An In Vitro Study. Mol. Neurobiol..

[131] Tondro G., Mohammadi A., Rajabzadeh G., Moradi H.R., Negah S.S. (2023). Niosomal Curcumin Inhibited Gliomagenesis-Related Markers in U87 Cell Line. Res. Sq..

[132] Jiang B., Yang Z., Shi H., Jalil A.T., Saleh M.M., Mi W.-Y. (2022). Potentiation of Curcumin-Loaded Zeolite Y Nanoparticles/PCL-Gelatin Electrospun Nanofibers for Postsurgical Glioblastoma Treatment. J. Drug Deliv. Sci. Technol..

[133] Zhang H., van Os W.L., Tian X., Zu G., Ribovski L., Bron R., Bussmann J., Kros A., Liu Y., Zuhorn I.S. (2021). Development of Curcumin-Loaded Zein Nanoparticles for Transport across the Blood–Brain Barrier and Inhibition of Glioblastoma Cell Growth. Biomater. Sci..

[134] Senturk F., Çakmak S., Kocum I.C., Gümüşderelioğlu M., Öztürk G.G. (2021). GRGDS-Conjugated and Curcumin-Loaded Magnetic Polymeric Nanoparticles for the Hyperthermia Treatment of Glioblastoma Cells. Colloids Surf. A Physicochem. Eng. Asp..

[135] Javed B., Zhao X., Cui D., Curtin J., Tian F. (2021). Enhanced Anticancer Response of Curcumin- and Piperine-Loaded Lignin-g-p (NIPAM-Co-DMAEMA) Gold Nanogels against U-251 MG Glioblastoma Multiforme. Biomedicines.

[136] Arzani H., Adabi M., Mosafer J., Dorkoosh F., Khosravani M., Maleki H., Nekounam H., Kamali M. (2019). Preparation of curcumin-loaded PLGA nanoparticles and investigation of its cytotoxicity effects on human glioblastoma U87MG cells. Biointerface Res. Appl. Chem..

[137] Maiti P., Plemmons A., Dunbar G.L. (2019). Combination Treatment of Berberine and Solid Lipid Curcumin Particles Increased Cell Death and Inhibited PI3K/Akt/mTOR Pathway of Human Cultured Glioblastoma Cells More Effectively than Did Individual Treatments. PLoS ONE.

[138] Maiti P., Scott J., Sengupta D., Al-Gharaibeh A., Dunbar G.L. (2019). Curcumin and Solid Lipid Curcumin Particles Induce Autophagy, but Inhibit Mitophagy and the PI3K-Akt/mTOR Pathway in Cultured Glioblastoma Cells. Int. J. Mol. Sci..

[139] Yeo S.-H., Kim M.J., Shim Y.K., Yoon I.C., Lee W. (2022). Solid Lipid Nanoparticles of Curcumin Designed for Enhanced Bioavailability and Anticancer Efficiency. ACS Omega.

[140] Wang Z., Wang X., Yu H., Chen M.-H. (2022). Glioma-Targeted Multifunctional Nanoparticles to Co-Deliver Camptothecin and Curcumin for Enhanced Chemo-Immunotherapy. Biomater. Sci..

